# Intrusion Detection in the Internet of Things: A Comprehensive Review of Techniques, Architectures, Datasets, and Emerging Trends

**DOI:** 10.3390/s26113405

**Published:** 2026-05-27

**Authors:** Asma Komal, Shuaiyong Li

**Affiliations:** 1 School of Computer Science, Chongqing University of Posts and Telecommunications, Chongqing 400065, China; asmakomal211@gmail.com; 2 Key Laboratory of Industrial Internet of Things and Networked Control, Ministry of Education, Chongqing University of Posts and Telecommunications, Chongqing 400065, China

**Keywords:** Internet of Things (IoT), intrusion detection system (IDS), anomaly detection, Explainable Artificial Intelligence (XAI), federated learning, lightweight IDS, TinyML, IoT security, benchmark datasets, deep learning in IoT, cybersecurity, adversarial attacks, network intrusion, validation strategies

## Abstract

As the Internet of Things (IoT) grows, strong, scalable, and adaptive intrusion detection systems (IDS) become increasingly critical for protecting IoT environments. This paper presents a comprehensive and systematic survey of IDS techniques for IoT environments, covering literature from 2021 to early 2026. The review introduces a multidimensional taxonomy that categorizes IDS approaches by detection strategy, learning paradigm, deployment architecture, and evaluation methodology. We examine conventional techniques, such as signature-based and anomaly-based detection, as well as modern machine-learning and deep-learning approaches. Furthermore, emerging paradigms, including Federated Learning, Explainable AI (XAI), TinyML, Large Language Models (LLMs), Transformer, Quantum Machine Learning, Generative Adversarial Networks and Incremental Learning, are analyzed with respect to their applicability to resource-constrained IoT environments. The paper also provides a detailed analysis of publicly available IDS datasets, validation protocols, and evaluation metrics used for benchmarking detection systems. In addition, critical challenges, including dataset realism, adversarial robustness, scalability, privacy preservation, and ethical considerations, are discussed. Finally, we highlight open research directions and propose guidelines for designing next-generation, trustworthy, and scalable IDS frameworks for IoT networks.

## 1. Introduction

The Internet of Things (IoT) has completely reshaped how we live and work, linking billions of smart devices across our homes, hospitals, factories, and cities. While this massive connectivity brings incredible convenience, it also creates a much larger playground for cyber attackers. The core problem is that most IoT devices are not built with heavy security in mind; they have limited memory, battery life, and processing power, and they often run in open or decentralized environments. Because standard security protocols are too heavy for these constrained devices, IoT networks are left highly vulnerable to a wide range of cyber threats [1,2].

To protect these networks, Intrusion Detection Systems (IDS) serve as a crucial first line of defense. Traditionally, we rely on two main approaches: Signature-Based IDS and Anomaly-Based IDS. SIDS works much like a traditional virus scanner, matching network traffic against a known list of attacks [3]. It is fast and accurate, but it completely misses zero-day threats. AIDS, on the other hand, learns what normal network behavior looks like and flags anything unusual. This helps catch new attacks, but it often triggers too many false alarms and requires massive amounts of labeled data, which is incredibly tough to manage on resource-constrained IoT devices [4,5]. In IoT environments, moreover, these traditional paradigms struggle with the performance and deployment challenges posed by resource scarcity. Recent improvements in artificial intelligence (AI) introduced powerful techniques to overcome these limitations. Over the years, machine learning and deep learning techniques such as support vector machines, convolutional neural networks, and ensemble classifiers have significantly enhanced accuracy of threat detection [6,7]. But these AI models come with their own set of problems. Many of them operate as opaque black boxes. When an AI system flags a threat, it does not always explain why. In high-stakes areas like smart agriculture or autonomous driving, lacking this transparency is a major safety risk [8]. The demand for transparent, immediate, and lightweight security solutions has spurred a new wave of research. However, existing literature [6,9,10] often provides a fragmented view of IoT security, typically focusing on a single dimension like ML algorithms, without reconciling these techniques with physical deployment constraints. Consequently, there remains no unified perspective that integrates detection strategies, deployment architectures, and emerging paradigms such as Incremental Learning and Quantum Machine Learning within a single analytical framework. As shown in Table 1, which compares recent studies from 2021 to early 2026, most reviews overlook this intersection, leaving a significant gap for research that balances performance, deployment feasibility, and ethical considerations.

### 1.1. Motivation, Scope, and Contributions

To address the aforementioned gaps, this review provides a comprehensive, integrated analysis of next-generation IoT IDS. The primary contributions of this paper are:

#### 1.1.1. Integrated Multi-Dimensional Taxonomy

Unlike traditional surveys that categorize IDS primarily by learning algorithms, this work proposes a novel taxonomy mapping detection strategy directly to system architectures (Edge, Fog, Cloud) and learning paradigms.

#### 1.1.2. Comprehensive Architectural Trade-Off

We systematically evaluate centralized, distributed, edge-based, fog-based, and hybrid architectures across five technical dimensions: latency, scalability, computational load, privacy preservation, and real-time response capability.

#### 1.1.3. Deep Dive into Emerging AI and Robustness

We extend beyond standard ML to analyze the integration of Federated Learning, Explainable AI, TinyML, LLMs, Transformer, QML, GANs and Incremental Learning are essential for handling constantly evolving threats.

#### 1.1.4. Critique of Validation and Benchmarking

We critically evaluate public IoT datasets and validation protocols, highlighting the necessity of cross-environment generalization to move models from benchmark testing to reliable field deployment.

#### 1.1.5. Roadmap for Future Research

This review identifies high-impact research directions, including ethical AI deployment, cross-dataset validation strategies, and the design of scalable, heterogeneous IDS ecosystems.

Recent literature from 2021 to early 2026 shows that IoT IDS research has expanded from conventional ML/DL approaches toward federated, explainable, lightweight, adaptive, and trustworthy IDS frameworks. Table 1 compares this review with recent IoT IDS surveys published from 2021 to early 2026, including newly added 2025–2026 studies recommended by the reviewers. The comparison shows that most reviews focus on specific techniques or architectures, whereas our study provides a comprehensive view across critical dimensions, thereby filling a gap in the literature.

### 1.2. Literature Selection Methodology

To ensure the thoroughness and integrity of our study, we conducted a systematic literature search covering literature published from 2021 to early 2026. We studied several important academic databases, including IEEE Xplore, ACM Digital Library, ScienceDirect, MDPI and SpringerLink. Preprints from arXiv were included only when no peer-reviewed versions were available. The search combined keywords such as IDS, “IoT security”, “machine learning IDS”, “deep learning IDS”, ”federated learning IoT”, “explainable AI IDS”, and “adversarial attacks IoT”. In total, 300 papers were initially screened. After removing duplicates and filtering for relevance to IoT-based IDS, 200 studies were retained and cited in this review. Of these, approximately 160–170 papers were examined in depth across three dimensions:

- Core ML/DL-based IDS models (summarized in Table 1),
- Emerging techniques such as federated learning, explainable AI, reinforcement learning, and blockchain.
- Datasets and benchmarking practices for IDS evaluation.

The rest of this paper is organized as Section 2 gives a full overview of IoT IDS approaches. Section 3 discusses several types of IDS architectures and deployment models. Section 4 presents a structured classification of IoT attacks and vulnerabilities. Section 5 discusses new AI paradigms, and Section 6 focuses on datasets and validation methods. In Section 7 and Section 8, we discuss both the Robustness and Generalization problems and potential solutions for further research. In Section 9, we summarize the key points of the work.

## 2. Taxonomy of IDS-IoT Techniques

**Figure 1:**
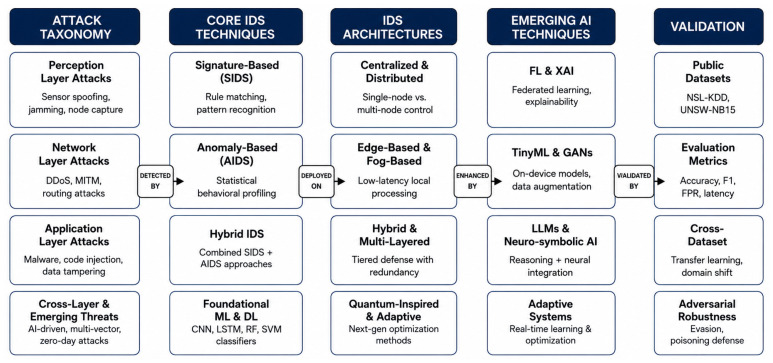
Proposed Holistic IDS-IoT Taxonomy and Pipeline.

Most Intrusion Detection Systems (IDSs) for the IoT are categorized by their primary detection strategy and learning methods. This classification shows how methods have evolved from signature-based to data-driven approaches, including their hybrid forms. Because IoT is continually evolving and has limited resources, it is necessary to evaluate the advantages and disadvantages of each method, including accuracy, computational overhead, and real-time adaptability. This review does not lie in proposing a new standalone IDS algorithm, but rather in providing an integrated, full-scope survey framework. As illustrated in Figure 1, our proposed multi-dimensional taxonomy conceptually differentiates this review from previous literature by mapping the complete IDS pipeline across five interconnected axes. Specifically, IoT attacks are detected via core IDS techniques, (deployed on targeted architectures, enhanced by emerging AI paradigms, and finally rigorously validated for real-world robustness. By connecting what is detected, where it is deployed, how it learns, and how it is evaluated, this framework addresses the fragmented nature of existing IoT security research.

### 2.1. Classification by Detection Approach

IDSs are primarily classified by their threat-detection methods. These include Signature-Based IDS, Anomaly-Based IDS, and Hybrid IDS.

#### 2.1.1. Signature-Based IDS

Signature-based IDS matches network traffic against known attack signatures. It is accurate for established threats and has low false-positive rates [28,29,30]. However, it cannot detect zero-day attacks or evolving threats, requires frequent updates, and struggles with encrypted payloads [19]. The need to manually update signature databases also imposes a maintenance burden; it is too slow and hinders IoT systems from adapting quickly to new threats. Some studies have explored the use of machine-learning filters to incorporate context-aware features [24,25], but the rigidity of SIDS makes it challenging to address emerging threats.

#### 2.1.2. Anomaly-Based IDS

Anomaly-based IDS, by contrast, learns normal behavior and flags deviations. It is effective for detecting unknown attacks, particularly in evolving IoT environments [31,32]. AIDS includes a wide range of methods that can be grouped into different categories:

- Statistical and Knowledge-Based AIDS: These techniques depend on predetermined thresholds and heuristics established by experts to identify anomalies [7,31].
- Machine Learning-Based AIDS: This sub-category uses supervised and unsupervised models, such as SVMs, Decision Trees, and K-Nearest Neighbors, for modeling normal behavior [32,33].
- Deep Learning-Based AIDS: This sophisticated method employs architectures such as CNNs, LSTMs, and Auto-encoders to discern complex temporal or spatial patterns within data [9,34,35,36].
- Reinforcement Learning (RL)-Based AIDS: RL-based systems, such as DQN and PPO, learn the best strategies to detect attacks in real-time, continuous environment [37,38].

AIDS methods are flexible and can detect zero-day attacks. Still, they often exhibit high false-positive rates, require large labeled datasets for supervised learning, and entail high computational overhead, making them difficult to deploy on IoT devices with limited resources.

#### 2.1.3. Hybrid IDS and Emerging Paradigms

To overcome the limitations of both SIDS and AIDS, Hybrid IDS architectures have gained significant traction. They typically implement a signature layer to quickly detect existing attacks and an anomaly-detection layer to detect new intrusions [32,39,40].

To synthesize these detection approaches from a practical engineering perspective, it is critical to evaluate their real-world deployment trade-offs. As highlighted in recent deployment-focused studies by [23], alongside foundational works [19,21], SIDS is highly feasible for resource-constrained edge deployment due to its low computational overhead. However, it poses a severe operational risk by failing against zero-day attacks and requiring continuous manual signature updates. Conversely, extensive evaluations by [25] and other researchers [26,41] demonstrate that AIDS and ML-based models adapt better to dynamic IoT traffic and concept drift. Nevertheless, they caution that these models demand periodic retraining and often suffer from high false-positive rates, leading to critical alert fatigue. To mitigate these extremes, lightweight hybrid architectures [14,42] offer a practical multi-layer defense, though they significantly increase the architectural complexity and maintenance burden for lightweight IoT nodes.

Recent research that uses new AI paradigms has further enhanced hybrid systems. Explainable AI methods, such as SHAP and LIME, make it easier to understand machine learning and deep learning models, which is critical in safety-sensitive domains such as healthcare IoT and autonomous systems, where trust, accountability, and regulatory compliance are essential. Additionally, FL allows IDS models to be collaboratively trained across distributed devices without sharing raw data, thereby preserving privacy. This is particularly valuable for sensitive environments like smart homes and wearable health monitors [4,43,44,45].

Table 2 presents a comparative analysis of these architectures across five technical dimensions: processing location, latency, scalability, privacy, and typical algorithmic techniques. While centralized models offer greater depth for complex tasks such as LLM integration, they incur high latency and pose privacy risks. Conversely, Edge-based and Hybrid models are emerging as the preferred choice for time-sensitive IoT applications due to their ability to process data locally and preserve user privacy through decentralized learning paradigms.

### 2.2. Classification by Learning Technique

Beyond their core detection strategy, Intrusion Detection Systems (IDS) are also defined by the specific learning paradigm they use. This distinction is critical because it highlights distinct trade-offs among computational cost, data requirements, and performance.

#### 2.2.1. Machine Learning Approaches

Many foundational anomaly-based IDS are built on traditional ML models that typically require careful, manual feature engineering to perform well. These are generally split into two categories. Supervised learning techniques require labeled datasets to classify network traffic as either benign or malicious [19,40]. Common algorithms such as SVM, KNN, and DT have demonstrated strong performance on benchmarks including the ToN-IoT and BoT-IoT datasets [19].

However, they often struggle with scalability and the intensive labor required for feature engineering [46,47]. In contrast, unsupervised learning methods are gaining traction due to the scarcity of labeled IoT data. These models learn patterns directly from unlabeled traffic to identify anomalies. K-Means clustering is a popular choice for its speed and simplicity. At the same time, more sophisticated methods, such as Gaussian Mixture Models (GMMs) and Singular Value Decomposition (SVD), can isolate more complex anomalies [46].

#### 2.2.2. Deep Learning Approaches

Deep Learning methods have transformed IoT intrusion detection by largely automating feature extraction [43,45]. This ability to learn features directly from raw data is a significant advantage over traditional ML, especially when processing large and complex datasets. Different DL architectures are better suited for specific tasks. For instance, CNNs are effective in extracting spatial or structured traffic features. Additionally, these systems are being redesigned to operate on devices with limited processing power, such as those at the network edge.

Meanwhile, RNNs and their variants, such as LSTMs, are designed for temporal sequences, making them well-suited for detecting slow-evolving attacks, such as botnet activity or gradual data exfiltration. Recent architectures, such as Transformers and attention-based networks, can capture long-range dependencies in traffic patterns. In some cases, they outperform LSTMs. Lastly, GANs [12,13] have two main applications: detecting anomalous patterns and generating synthetic data to expand augmented training sets. However, they can be hard to train and understand [48].

Table 3 gives a brief overview of these important deep learning architectures, including their pros and cons and how they can be used in IoT intrusion detection.

## 3. IDS Architectures and Deployment Models

Because the IoT is decentralized and resource-constrained, traditional centralized security models are ineffective. As a result, the architecture of intrusion detection systems (IDSs) has evolved to meet the needs of diverse IoT environments. This section covers the most common IDS architectural paradigms and focuses on their main ideas, trade-offs, and optimal use cases. The focus is on the shift from monolithic, centralized models to more flexible, multi-layered architectures, with an emphasis on balancing latency, computational load, and scalability.

**Figure 2:**
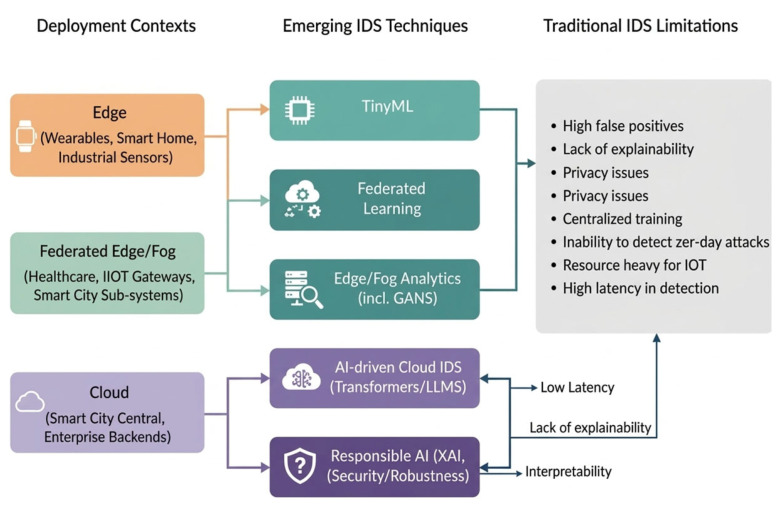
Integration of emerging IDS techniques across IoT deployment tiers to resolve traditional security limitations, highlighting XAI as a technical interpretability component within the broader Responsible AI governance framework.

To fully conceptualize this architectural shift, Figure 2 illustrates how modern IDS techniques are mapped to specific IoT environments to overcome the limitations of traditional security models. Starting at the device level, highly constrained edge environments (such as wearables and smart home sensors) use TinyML for fast, localized anomaly detection [14,15]. Moving up to the federated edge and fog layers, systems rely on Federated Learning [11,55] and on local analytics such as GANs [12,13]. This allows different networks to collaboratively train security models without ever exposing sensitive raw data. At the top, the cloud layer handles computationally heavy tasks, using powerful AI models like Transformers and LLMs to analyze global threat intelligence and catch complex zero-day attacks [20,56].

Together, these targeted approaches help eliminate traditional bottlenecks, such as slow response times, privacy risks, and single points of failure. Furthermore, to address the ethical deployment of these systems, the figure outlines the critical relationship between Responsible AI and Explainable AI. It is important to make a clear distinction here: Responsible AI serves as the broad, overarching governance framework that ensures fairness, privacy, accountability, and system robustness [57]. XAI, conversely, is a specific technical component within that framework, designed to make the AI’s complex decisions transparent and understandable to human operators [21,58]. By bringing together these deployment tiers, advanced algorithms, and ethical guidelines, Figure 2 presents a complete, next-generation IoT IDS pipeline.

### 3.1. Centralized and Distributed Architectures

Previously, IDS architectures were largely centralized, with a single, high-capacity node, such as a cloud server, that collected and processed all network traffic. This architecture provides substantial processing power and a global view of the network, making it effective for long-term threat analytics and complex, resource-intensive analyses [59,60]. However, centralized architectures suffer from major drawbacks: a single point of failure, high communication delays, and bandwidth congestion. To address these drawbacks, distributed architectures were introduced. They reduced centralization in the detection process by deploying lightweight IDS agents across various IoT nodes and network segments. This design improves scalability and resilience in large environments, such as vehicular networks and IIoT. The main challenge is the additional work associated with inter-agent communication, model synchronization, and maintaining consistency across evolving network topologies [61].

### 3.2. Edge-Based and Fog-Based Architectures

To address the latency and bandwidth issues associated with centralized models, modern architectures have shifted computation closer to the data [62]. Edge-based IDS puts intrusion detection logic right on edge devices or local gateways [63]. This enables real-time responses and significantly reduces the need to send raw data to a central server [64]. This model is most suitable for applications that require immediate action, such as smart homes and real-time health monitoring. However, edge-based systems face challenges because edge nodes lack substantial processing power and may struggle to detect complex, coordinated attacks that affect multiple devices. Fog-based architectures offer a more balanced solution by adding a layer of processing between the edge and the cloud [64,65]. This fog layer aggregates data from multiple edge devices, allowing mid-latency analytics and contextual intelligence. It strikes a balance between rapid responses and comprehensive network visibility, making it suitable for fields such as industrial automation and precision agriculture [64].

### 3.3. Hybrid Edge–Fog–Cloud IDS and Deployment Granularity

**Figure 3:**
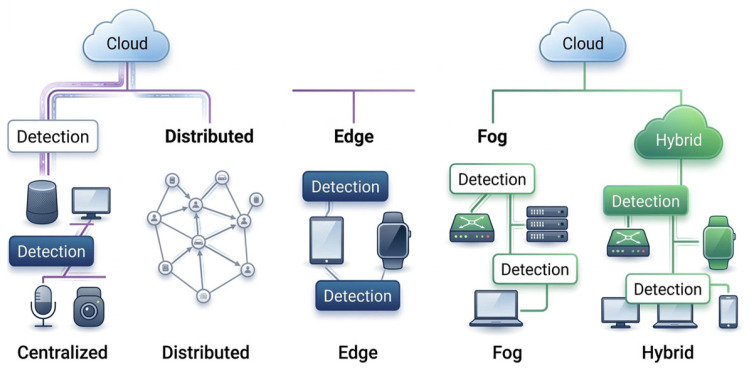
IDS Architectures.

Relying on a single architectural layer often forces a compromise between fast response times and deep analytical capabilities. To get the best of both worlds, hybrid models bring the edge, fog, and cloud layers together into one cohesive system. As illustrated in Figure 3, these IDS architectures can be broadly categorized based on their deployment strategy and analysis techniques. By intelligently splitting up the detection workload across these levels, multi-layered architectures strike a much better balance in terms of speed, accuracy, and resource utilization [66,67]. Research highlights that such systems adjust dynamically to diverse threats while efficiently managing trade-offs in energy and network usage.

In a typical real-world setup, lightweight TinyML models sit right at the edge to catch anomalies in real-time, ensuring an immediate response [68,69]. If something looks suspicious, it gets passed up to the fog gateways for a broader, contextual review. Meanwhile, the heavy lifting like spotting long-term trends or retraining massive models is left to the cloud. However, making this work outside the lab means we have to be very practical about the actual hardware limits of IoT devices and how efficiently we train them [21,23]. Adding federated learning into the mix is also a significant advantage, as it lets distributed nodes collaborate and learn without exposing sensitive data to a central server.

Table 4 breaks down these different deployment strategies, highlighting their main strengths and limitations, the techniques that suit them best, and where they actually fit within the IoT ecosystem.

## 4. Taxonomy of Layer-Wise Attacks and IoT Security

As IoT intrusion detection strategies continue to improve, it is essential to understand the full attack surface. Because IoT environments are highly heterogeneous, threats must be examined layer by layer. This section presents a systematic list of attacks that can occur at the three main layers of the IoT architecture: Perception, Network, and Application [70]. It also discusses advanced threats that are difficult for traditional defenses to address [71]. This organized approach is important for designing IDS models that are aware of their environment and specific to each layer [72].

### 4.1. Perception Layer Attacks

The Perception Layer comprises physical components such as sensors, actuators, and microcontrollers that interact with the real-world [70]. This layer provides direct sensing and actuation functions. This layer is the most vulnerable because it is directly exposed, and these devices often lack robust built-in security [73]. Attackers exploit these weaknesses in several ways. They may gain physical access to extract cryptographic keys or tamper with device functions [74,75]. Fake nodes can be deployed to send misleading data, posing serious risks to critical infrastructure [72]. Other common threats include Replay attacks [76], where valid data is recorded and sent back to the system to trick it [71], signal jamming, which disrupts communication [75], and passive eavesdropping on non-encrypted channels to steal sensitive information.

It is difficult to protect these low-power, resource-limited devices because traditional, centralized Intrusion Detection Systems (IDSs) are too resource-intensive to run. But a new generation of smart, lightweight defenses is emerging to address this. For example, researchers are now using small, efficient on-device anomaly-detection models directly on microcontrollers to detect signal tampering in real time [26]. Other targeted solutions are using unique radio-frequency (RF) fingerprinting to find real devices [77], using lightweight authentication protocols that do not need much computing power, and hardware-level solutions like Physically Unclonable Functions (PUFs) to create tamper-proof identities [78].

### 4.2. Network Layer Attacks

The network layer of the IoT is a complex system that governs data transmission between devices, gateways, and the cloud [76]. Because it is based on protocols and serves as the primary path for data flow, it is a prime target for attacks that aim to modify network topology or overload communication channels. Attackers exploit common threats to disrupt this flow. Some might use Sybil [79], and sinkhole attacks [80], where a single malicious device takes on multiple identities to break the network’s agreement, or a malicious node offers an optimal route to trick traffic into following it and then drops or changes it. Others might employ selective forwarding and Wormhole Attacks to drop important data packets [81], or build hidden tunnels between cooperating nodes to distort the network topology.

We also observe large-scale pile-ups in the form of DoS attacks, often executed by botnets such as Mirai, which send a large volume of traffic to networks, making them too busy to provide services [82,83]. Traditional network-based IDSs and standard security systems struggle to handle these new threats due to routing behaviors and encrypted traffic, which create dangerous blind spots [81]. To address these constraints, sophisticated methodologies are being implemented. Federated Learning, for example, lets one train models in different places without giving up the privacy of one’s raw data [84]. Graph Neural Networks (GNNs) are also increasingly used to detect complex, topology-based attacks [85].

### 4.3. Application Layer and Advanced Threats

The Application Layer is the control center of an IoT system. It covers everything from user interfaces and mobile apps to the backend logic that enables high-level services. This makes it highly attractive for attackers targeting software and user trust. Common threats include code injection in smart assistants and apps [86]. Attackers also use insecure APIs or malware and ransomware to fully take control of devices [87,88,89]. Beyond direct code exploits, attackers employ social engineering techniques, such as phishing, to obtain passwords [90], and smart side-channel attacks that analyze hardware emissions, such as power consumption, to steal private information [91].

This layer is often difficult for traditional intrusion detection systems to handle because they are too rigid to accommodate the fast-changing nature of these attacks [92]. Modern defenses are shifting toward adaptability. For instance, SHAP and LIME are XAI tools used to clarify security alerts. Instead of receiving a generic alert, an analyst can quickly understand why the model flagged an item as suspicious. This builds trust and speeds up response times [93]. GANs also work like a digital sparring partner by making realistic, fake attack traffic that defense models can use to practice against threats they have never seen before. This makes them much stronger and faster [94].

### 4.4. Cross-Layer and Emerging Threats

Some of the most dangerous threats to IoT systems do not remain within a single layer and propagate across the entire system, which means we need defenses that are equally complex and multifaceted. These emerging threats include Adversarial Machine Learning, where attackers make tiny, almost invisible changes to data to fool an AI-based IDS into ignoring a real threat [95,96]. We also face Zero-Day and multi-stage attacks, which are complex, slow-evolving campaigns that exploit unknown vulnerabilities and unfold across multiple system layers over time [97].

Additionally, Insider Threats or malicious activity by trusted users are very hard to detect and require a hybrid of machine learning and logical reasoning [14]. Finally, the quiet theft of sensitive data through Privacy Violations is now being countered by advanced privacy-preserving technologies like Secure Multi-Party Computation (SMPC) [98] and Federated Analytics. These advanced threats demonstrate the urgent need for next-generation security models. For instance, Transformer-based AI is proving to be a game-changer in cybersecurity by helping to find complex, multi-stage attacks by learning temporal dependencies across event sequences between seemingly unrelated events over time. Similarly, neuro-symbolic AI offers a powerful approach by blending the pattern-recognition strengths of neural networks with the clear logic of rule-based systems, making it highly effective at spotting insider threats.

**Figure 4:**
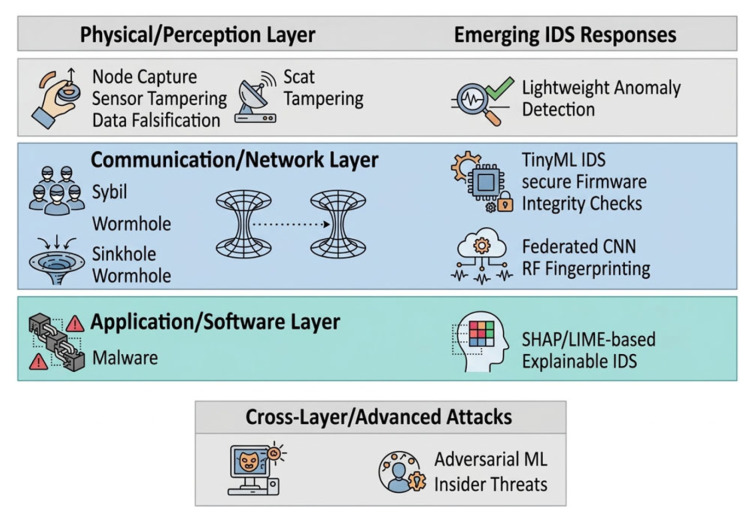
IoT layer-wise Attack Taxonomy and its Emerging IDS Responses.

A clear understanding of this taxonomy is essential, as it exposes the limitations of older security models. By mapping each layer’s vulnerabilities to modern, specialized so5lutions, researchers can develop the robust defenses required for future evolving IoT landscape. Table 5 summarizes this taxonomy by mapping specific attack types directly to their corresponding IDS design implications and reported performance metrics. Figure 4 illustrates the IoT layer-wise Attack Taxonomy and reported performance of these emerging responses.

## 5. Emerging AI Techniques in IoT IDS

**Figure 5:**
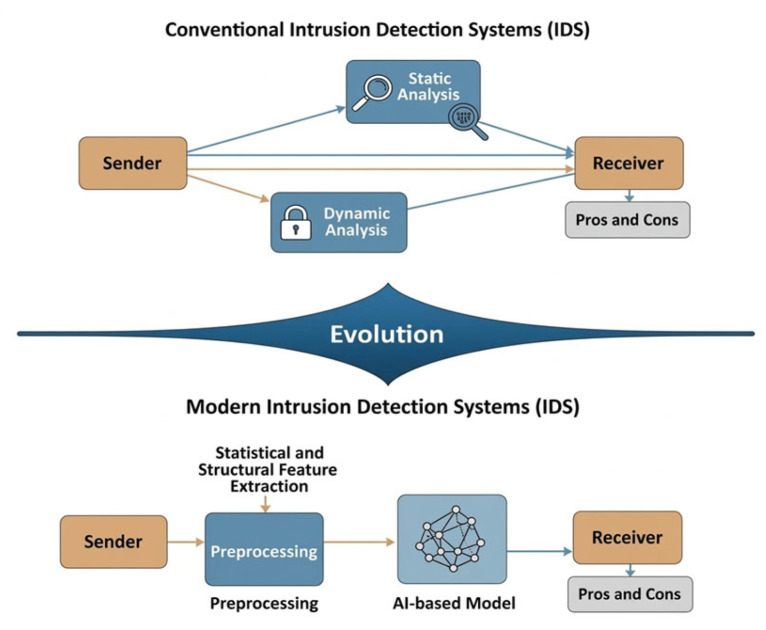
Evolution of IDS Approaches.

Traditional IDS methods are still widely used, but it is becoming clearer that they do not work as well against modern IoT threats. The attack surface is expanding exponentially, and IoT-based threats are becoming more complex, larger, and more dynamic. This means that traditional solutions are no longer enough. New AI methods are revolutionizing how we solve security problems in IoT, making our defenses more accurate, scalable, and private. This section provides a comprehensive overview of these advanced methods, detailing their core concepts, key contributions, and the challenges they face in securing IoT environments. As illustrated in Figure 5, we have moved beyond older, rule-based Intrusion Detection Systems (IDS) that relied on rigid static and dynamic analysis. While the emerging paradigms innovative solutions to IoT security challenges, evaluating their practical efficacy requires a closer look at their empirical results. As highlighted by several recent comprehensive surveys in 2025 and 2026, comparing these methodologies is inherently complex due to the diverse environments and highly variable constraints of IoT deployments (e.g., edge vs. cloud). To provide a concrete perspective on the current state-of-the-art, Table 6 synthesizes the reported performance metrics and resource implications of these representative IoT IDS methods. This comparison highlights not only the detection capabilities (such as accuracy and F1-scores) but also the critical trade-offs involving computational latency, communication overhead, and architectural limitations, which are essential for practical, deployment-aware evaluations.

### 5.1. Federated and Explainable AI for Trustworthy IDS

As IoT environments become increasingly distributed, privacy-sensitive, and heterogeneous, conventional intrusion detection models face a growing need for decentralized and trustworthy solutions. This has catalyzed the integration of two key paradigms: Federated Learning and Explainable AI.

#### 5.1.1. Federated Learning for Privacy-Preserving Detection

Federated Learning is a decentralized approach that enables IoT nodes to collaborate in training a global IDS model without sharing their raw data [113]. Only model updates are sent, thereby keeping data private and reducing communication overhead [114]. This model is well-suited to IoT systems where privacy regulations (e.g., GDPR) and slow internet connections pose significant challenges [115]. Recent research has shown that FL-based IDS can perform well across a variety of settings, achieving high accuracy [116]. Researchers have also examined how to improve FL performance on non-IID (non-independently and identically distributed) data. This is a common problem in IoT because each device may act differently [117].

Researchers are also combining FL with other technologies, such as blockchain [104] for enhanced security and Transformers for improved long-range pattern detection, to address issues such as data imbalance and model drift. Nevertheless, FL faces persistent hurdles, including unevenly distributed data, device dropout, and synchronization latency, which can slow the convergence of the global model [42,100,117].

#### 5.1.2. Explainable AI for Transparent Decisions

AI-driven IDS has demonstrated exceptional performance; however, its black-box characteristics raise significant concerns in safety-critical sectors such as healthcare and industrial control, where trust and accountability are essential. Explainable AI mitigates this by providing users with understandable information about how a model makes decisions [118,119]. SHAP and LIME are two methods that show which features led to an alert. This helps human administrators check alerts and fix false positives [120,121]. Researchers have combined XAI with IoT-specific models to demonstrate how network activity changes over time and to increase user trust in federated settings. XAI can slow down computers, but the benefits of more trust, compliance, and auditability make it an important part of modern IDS [122,123,124,125].

The integration of FL and XAI reflects a broader trend toward decentralized, interpretable, and user-centered security solutions.

#### 5.1.3. Responsible and Explainable AI by Design

In safety-critical IoT environments, explainability and ethical governance must be foundational design requirements rather than post hoc additions. While Explainable AI technically interprets model decisions through feature attribution or visual aids, Responsible AI serves as a broader governance framework encompassing fairness, accountability, privacy, and human oversight [111,126,127]. An IDS might achieve high accuracy, but it remains un-deployable if it produces biased alerts, exposes sensitive data, or lacks transparent audit trails [58,118,119,120,121,122,123,124,128,129].

Therefore, these requirements must be embedded natively across the entire IDS lifecycle from dataset collection to distributed update cycles. Supporting this design-first paradigm, recent research emphasizes that XAI-enabled IoT IDSs must be strictly evaluated on the trade-off between detection accuracy, computational overhead, and explanation quality [21].

To guide future implementations, Table 7 maps these Responsible AI and XAI requirements to their respective design phases, ensuring that transparency is built into the system by default.

### 5.2. Resource-Aware and Adversarial Techniques

Beyond privacy and interpretability, the limitations of traditional IDS in handling resource constraints and evolving threats have been addressed by two complementary approaches: TinyML for on-device detection and GANs for adversarial robustness.

#### 5.2.1. TinyML for On-Device IDS

Traditional IDS models often require substantial memory, processing power, and energy, making them unsuitable for low-power IoT microcontrollers [15]. TinyML has come up with a solution to this problem by putting optimized machine learning models (usually less than 1 MB) directly on devices that do not have many resources [14]. This localized inference enables real-time threat detection, reduces latency, and preserves data privacy by eliminating the need for continuous communication with the cloud [133]. Key techniques such as model pruning and quantization enable model compression to fit within the tight memory and energy budgets of edge devices [132]. TinyML-based IDSs have limitations, however, because they cannot train on-device effectively and can be physically tampered with or reverse-engineered [134,135,136].

#### 5.2.2. Generative Adversarial Networks for Enhanced Robustness

Traditional IDS models have difficulty detecting rare or zero-day threats as attack methods become more stealthy and data-driven. Generative Adversarial Networks (GANs) counter this by generating realistic synthetic attack samples, augmenting small datasets, and improving IDS resilience [137]. This makes models less likely to avoid attacks and improves their performance against threats [138,139]. Conditional GANs (CGANs) and Autoencoder-GAN hybrids (AE-GANs) are two types of GANs that have been used to simulate attacks targeting specific protocols and to capture subtle anomalies in time-series telemetry. However, GANs require substantial computational resources and can be unstable during training, which makes them difficult to deploy on edge devices with limited resources [13,139,140,141,142]. Table 8 shows some examples of how GAN-based intrusion detection systems can be used in IoT environments, along with their best features and performance.

### 5.3. Advanced Behavioral and Trustworthy AI

Recent advances in natural language processing and hybrid reasoning are reshaping IDS design, enabling more contextual, interpretable, and ethically aligned detection frameworks.

#### 5.3.1. LLMs and Transformers for Contextual Intelligence

The introduction of Transformers and LLMs has facilitated a significant shift toward behavioral analysis in IoT security. These models are better than traditional RNNs and LSTMs at spotting multi-stage attacks or subtle changes in behavior over time because their attention mechanism lets them capture long-range dependencies in data sequences [56,143]. LLMs further assist analysts by describing anomalies in natural language, improving human-AI collaboration [144]. Although computationally intensive, model compression techniques are increasingly feasible for edge deployment [145]. Table 9 summarizes practical implementations and results of Transformer-based IDS, underscoring their contextual intelligence.

#### 5.3.2. Neuro-Symbolic AI and Responsible AI for Ethical Security

Neuro-symbolic AI combines the pattern-recognition power of neural networks with the clear reasoning of symbolic logic to fill the gap between deep learning models that do not work well and symbolic systems that are too fragile [126]. This mixed-method approach not only identifies anomalies but also provides logical explanations that are understandable to people [110]. Neuro-symbolic models are effective at detecting insider threats and complex, multi-stage attacks that violate the rules. Table 10 compares neuro-symbolic, neural, and symbolic IDS, outlining their relative strengths and limitations.

The broader field of Responsible and Ethical AI emphasizes the need for AI-driven IDS to be fair, open, and accountable, particularly in sensitive areas. This means ensuring that models are fair, that decisions can be audited, and that data privacy is protected [57,127]. Legal frameworks such as the EU AI Act are promoting the integration of these principles, which is necessary for security systems that are both trustworthy and compliant with the law. Table 11 shows the main ideas behind Responsible AI, how they can be used, and the problems that come with using them in IoT IDS.

The transition from conventional to novel AI methodologies in IoT Intrusion Detection Systems represents a significant shift from reactive, performance-oriented frameworks to proactive, comprehensive solutions that address a broader range of security and ethical issues. Table 12 offers a succinct comparative overview of both traditional (Signature-Based and Anomaly-Based) and significant emerging techniques, emphasizing their respective merits and demerits. This thorough comparison highlights the trade-offs inherent in developing robust and flexible intrusion detection systems across diverse IoT environments. It also suggests that the future of IoT security lies in integrating these ideas into smart, hybrid, and trustworthy defense systems.

### 5.4. Adaptive and Quantum-Inspired Emerging IDS Directions

Most traditional ML models for IDS assume that the network traffic they see during training will look the same during testing. But in the real world, IoT networks are always changing whether from firmware updates, adding new devices, or attackers shifting their strategies. This mismatch creates concept drift $P_{t}\left(X,Y\right)\neq P_{t+\Delta }\left(X,Y\right)$, meaning models that performed perfectly in the lab often fail quickly in actual deployment. To fix this, researchers are turning to incremental and continual learning. Instead of retraining a model completely from scratch (which is too expensive for resource-limited edge devices), these methods let the model learn on the fly from new traffic patterns. This is incredibly useful for catching zero-day attacks and adapting to unseen devices. For instance, recent work on incremental contrastive learning [147] helps models handle sparse and dynamic network data. Likewise, incremental federated learning [148] keeps models accurate over time without compromising privacy [113,114,116]. Still, these adaptive methods are not perfect; we have to be careful they do not suffer from catastrophic forgetting (losing knowledge of older attacks) or get tricked by poisoned data updates, though integrating blockchain can help secure this update process [149].

To break this down clearly, Table 13 provides a comprehensive summary of key adaptability issues, suitable learning approaches, and their remaining risks in evolving IoT environments.

Parallel to adaptive learning, QML is gaining attention as a futuristic, long-term paradigm for IoT IDS. Theoretically, QML offers significant advantages for high-dimensional optimization and rapid pattern recognition through quantum kernel methods, which could exponentially accelerate the detection of complex, multi-stage cyberattacks [150]. However, this emerging direction must be approached with caution. Currently, QML remains in its absolute infancy and is severely constrained by Noisy Intermediate-Scale Quantum (NISQ) hardware, limited qubit availability, and substantial computational overhead required for classical-to-quantum feature encoding [151]. Furthermore, there is a complete absence of realistic, IoT-scale quantum benchmarks to validate these theoretical models. Thus, while QML represents a promising frontier for next-generation IDS, it requires substantial hardware breakthroughs before it can be practically integrated into resource-constrained edge-fog-cloud architectures.

To wrap up our discussion on these emerging AI paradigms, it is important to look beyond just what they can do and instead focus on the real-world trade-offs they bring to the table. For instance, while highlighting how Federated Learning (FL) keeps data private, we cannot ignore the fact that it also introduces new risks like model poisoning and heavy synchronization delays, especially in unpredictable IoT environments [114,115]. Similarly, for Explainable AI (XAI), research [21] shows it makes models more trustworthy, using tools like SHAP or LIME often slows things down so much that they might not even run on small edge devices. In terms of local security, demonstrate that TinyML is great for avoiding cloud-related lag, but this comes at the cost of very limited model capacity and the headache of updating those devices in the field [14,15]. Furthermore, while GANs effectively address class imbalance, and LLMs offer superior contextual reasoning, they introduce risks of synthetic data bias and hallucination, alongside massive compute requirements. Finally, as noted by [26], adaptive paradigms like incremental learning are essential for handling concept drift, yet they remain susceptible to catastrophic forgetting and poisoned updates, necessitating the cautious, context-aware deployment strategies detailed in this survey.

## 6. Public Datasets and Validation Methods for Detecting Intrusions in the Internet of Things

The dependability, precision, and resilience of Intrusion Detection Systems (IDS) in IoT settings are fundamentally connected to the caliber of datasets and the stringency of evaluation protocols. Due to practical limitations in collecting diverse, labeled, and privacy-preserving IoT traffic data, researchers have increasingly relied on publicly available datasets to simulate attack scenarios and evaluate model performance. This section presents a systematic review of these benchmarks, distinguishing legacy collections from modern IoT-specific collections. It also examines the metrics and protocols required for fair and reproducible IDS validation.

### 6.1. The Change in Datasets: From Old to IoT-Centric

Earlier, IDS research relied heavily on older datasets such as DARPA 1998, KDDCUP 99, and NSL-KDD. These benchmarks provided structured, tabular, or packet-based datasets for supervised learning models. However, their relevance to IoT has largely faded because the attack types they employ are no longer effective, and there are no IoT-specific protocols or device-level heterogeneity [31,152,153,154]. They are not very useful for showing the modern, changing threat landscape.

To properly train and test modern security systems, we need data that looks like the real world. While older datasets were foundational, they often do not reflect today’s complex IoT environments.

**Figure 6:**
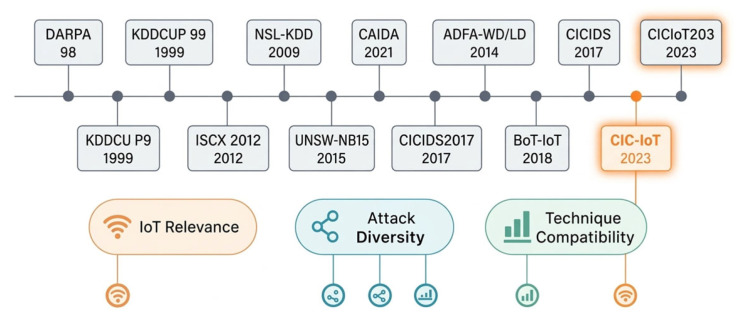
Timeline and Feature Evolution of IDS Datasets (1998–2023).

As shown in Figure 6, IDS datasets have progressively advanced from 1998 to 2023 in terms of complexity and feature diversity.

#### 6.1.1. Legacy Datasets: The Foundation

Many early intrusion detection systems were built using what are now considered legacy datasets. While historically important, they were created before the IoT explosion and lack the specific context required to train modern defenses. The lineage starting with DARPA 98 and its derivatives, KDDCUP 99 and NSL-KDD, provided the foundational benchmarks for early research. However, they are synthetic, contain outdated attack types, and do not reflect IoT environments [41,153,154,155,156]. Other valuable datasets, such as CAIDA, ISCX 2012, UNSW-NB15, and CICIDS2017 [157], provide realistic, large-scale network traffic, making them useful for studying general threats such as DDoS attacks. Still, they lack the specific device telemetry and IoT protocol data needed for specialized security systems [158]. Similarly, datasets such as ADFA-WD/LD are useful for analyzing host-level logs in Linux systems but are not IoT-centric.

#### 6.1.2. Modern IoT-Specific Datasets

To address this critical gap, a new generation of datasets has emerged that more accurately reflects the complexity of real-world IoT environments. These collections provide multi-protocol traffic and telemetry data from various devices, as well as up-to-date attack scenarios, making them essential for developing robust security solutions.

- BoT-IoT: This dataset focuses specifically on smart home environments and includes common attacks like DDoS, reconnaissance, and data theft. It is widely used for testing lightweight IDS models but is characterized by severe class imbalance, which can pose a challenge during training [159].
- TON-IoT: Offering a more diverse simulation, TON-IoT covers industrial, home, and office settings. Its key strength is its multi-modal nature, combining network traffic with device telemetry and system logs. This makes it ideal for advanced research in federated and transfer learning, though it requires significant preprocessing [160,161]
- CICIoT2023: As one of the most recent contributions, CICIoT2023 includes a wide array of modern threats like spoofing, malware injection, and even adversarial ML samples. Its rich and relevant data makes it indispensable for evaluating cutting-edge, AI-based security models [162,163,164].

### 6.2. Limitations and Gaps in Existing Datasets

Existing datasets for intrusion detection systems (IDS) in IoT environments still have significant limitations, which hinder the effectiveness of the models they are used to train. These datasets often exhibit synthetic bias, whereby simulated traffic fails to capture the true complexity of real-world network conditions. Furthermore, there is a general lack of diversity, as they do not adequately represent the wide range of IoT devices and deployment scenarios. Manual or heuristic labeling introduces labeling issues, resulting in noise and errors. In federated learning applications, datasets often exhibit non-IID (non-independent and identically distributed) distributions that do not reflect the heterogeneous nature of real-world data across devices.

Additionally, many datasets lack sufficient examples of advanced threats such as zero-day and adversarial attacks, making it difficult to train robust models; these gaps directly impact the generalizability of models and the reproducibility of research. Models trained on outdated or synthetic data often perform poorly when deployed in real-world settings. Models trained on outdated or synthetic data often perform poorly when deployed in real-world settings. As a result, emerging techniques such as FL-CNN, IoT-BERT, and Symbol-Net-ID increasingly rely on modern, context-rich datasets, including CICIoT2023 and TON-IoT, to evaluate their effectiveness under realistic conditions [144,160,161,162].

### 6.3. Dataset Realism and Generalization Risk

A major roadblock in evaluating IoT IDS is our continued reliance on datasets that are simply too old, synthetic, or imbalanced to represent real-world networks. Legacy datasets like KDDCUP99 and NSL-KDD are great as historical baselines, but they completely miss the mark for modern IoT; they do not capture IoT-specific protocols, diverse edge devices, or today’s complex attack behaviors [153,155,156].

Moving a step up, BoT-IoT gives us a more IoT-centric benchmark by mixing normal traffic with botnet attacks. However, because it relies on simulated environments and suffers from severe class imbalance, using purely accuracy to judge a model on this dataset often leads to dangerous overestimations of its reliability [159,165]. On the more realistic end, TON-IoT offers a richer, multimodal challenge by fusing network traffic, telemetry, and operating system logs across both IoT and IIoT setups [160,161].

Finally, CICIoT2023 really pushes the boundaries of modern benchmarking. It simulates 33 different attacks across a massive topology of 105 IoT devices, covering everything from DDoS and Reconnaissance to Mirai botnets [162,164]. Because of this extreme variety, future IDS research must move past basic accuracy. To prove a model is truly ready for deployment, we need to evaluate it using these modern datasets alongside time-aware and cross-device splits, focusing strictly on robust metrics like macro-F1, false positive rate, latency, and resource consumption [130,166,167].

To break this down clearly, Table 14 provides a critical look at dataset realism, typical attack topologies, and the best validation strategies across prominent IoT benchmarks.

### 6.4. Evaluation Metrics and Validation Protocols

To ensure fair and reproducible comparisons, IDS models must be evaluated using a consistent set of metrics and validation protocols specifically tailored to the IoT context.

#### 6.4.1. Key Evaluation Metrics

While raw Accuracy is a common metric, it can be misleading in scenarios with severe class imbalance, where a model that simply predicts the majority class can achieve high scores. Therefore, metrics that account for this are crucial:

- Precision:$\;P=\frac{TP}{TP+FP}$

The proportion of true positive predictions among all positive predictions.

- Recall (Sensitivity):$\;R=\frac{TP}{TP+FN}$

The proportion of true positives correctly identified among all actual positives.

- F1-Score: $F1=2\times \frac{Precision\times Recall}{Precision+Recall}$

The harmonic mean of precision and recall provides a balanced measure highly useful for imbalanced datasets.

- False Positive Rate (FPR):$\;FPR=\frac{FP}{FP+TN}$

The rate at which the IDS incorrectly flags benign traffic as malicious. A low FPR is critical for user trust and avoiding service disruption.

- AUC-ROC: The Area under the Receiver Operating Characteristic curve provides a comprehensive performance measure across all classification thresholds, making it robust to class imbalance [32,152].
- Confusion Matrix: In highly imbalanced IoT datasets, reviewing the confusion matrix is critical. Instead of relying on a single accuracy score, it visually breaks down the exact distribution of True Positives, True Negatives, False Positives, and False Negatives, providing essential transparency into an IDS model’s real-world reliability.

#### 6.4.2. Validation Protocols

The chosen validation protocol significantly impacts the reliability of the results:

- Holdout Validation: Splits the dataset into a training and testing set (e.g., 80:20). It is computationally efficient but can be sensitive to the specific data split.
- Partitions the dataset into k subsets, training on k−1 folds and testing on the remaining one, which reduces variance and provides a more robust estimate of performance [167]. The average accuracy across folds is a key indicator:

$$CVAccuracy=\frac{1}{k}\sum_{i=1}^{k}Accuracy_{i}$$

- Leave-One-Out Cross-Validation (LOOCV): Each instance is used once for testing. It is exhaustive but computationally impractical for large IoT datasets [130].
- Time-Based Validation: A protocol essential for sequential IoT traffic, where the model is trained on past data and tested on future data. Combining these protocols with context-aware metrics is essential for validation and crucial for federated settings, as it evaluates a model’s ability to generalize to new, unseen devices.

**Figure 7:**
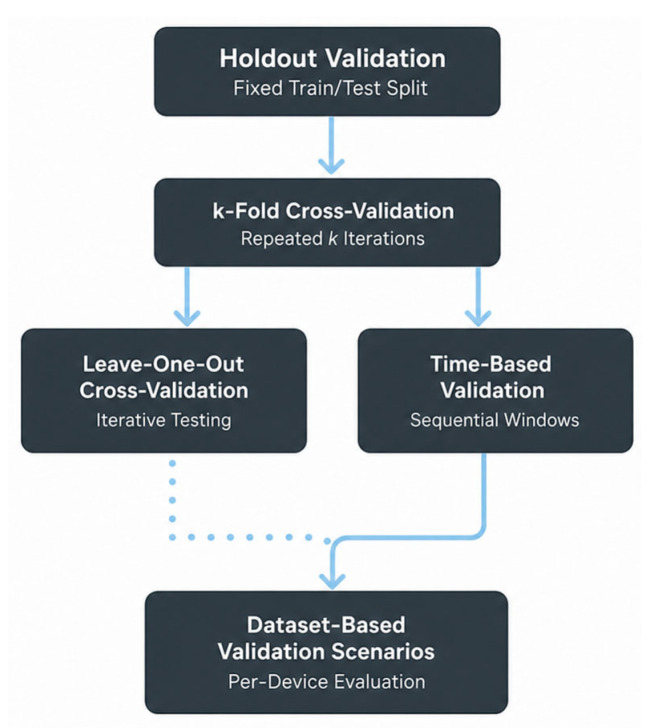
Protocol Flow for IoT-Specific IDS Validation Methodologies.

The overall protocol flow for IoT-specific IDS validation methodologies is shown in Figure 7.

#### 6.4.3. Recommended Validation Protocols for Reliable IoT IDS Evaluation

To move beyond high-level descriptions, it is crucial to apply practical, case-study-based insights when selecting datasets for IoT IDS evaluation. Historical baselines like KDDCUP99 and NSL-KDD [153,155] are severely outdated and should no longer be used as the sole evidence of modern IoT IDS performance. While UNSW-NB15 [158] remains a useful general network benchmark, it lacks specific IoT threat contexts. For targeted IoT evaluations, datasets like BoT-IoT [165] are highly effective for high-volume DoS and botnet scenarios, though researchers must be cautious of class imbalances. Conversely, TON-IoT [161] offers a much stronger multimodal benchmark by combining telemetry, logs, and network traffic, making it ideal for cross-environment testing. More recently, CICIoT2023 [162,164] provides a massive, modern attack landscape suitable for time-aware validation, while Edge-IIoTset [161] is perfectly tailored for device-level and edge-oriented testing.

Furthermore, random holdout evaluations fail to capture the temporal and heterogeneous nature of IoT traffic. Cross-dataset validation, chronological time-aware splits, and device-level testing are essential to prove a model’s generalization against concept drift and unseen devices [41,154,163]. For edge or TinyML deployment, latency, memory footprint, and computational overhead must also be strictly reported.

Recent literature provides practical examples of these rigorous strategies; for instance, [169] utilized a time-based holdout on the CICIoT2023 dataset to simulate temporal stream deployments, while [161] applied device-level CV on the Edge-IIoTset to verify cross-device generalization. Similarly, advanced ensemble models increasingly rely on hybrid 10-fold CV combined with holdout testing to ensure robustness across traffic variations [167,170], alongside NS-3 simulated environments to test contextual anomaly detection against novel attacks [128].

For edge or TinyML deployment, latency, memory footprint, and computational overhead must also be strictly reported alongside these protocols. To synthesize these best practices and guide future research toward practical robustness, Table 15 provides a definitive framework for IoT IDS validation.

### 6.5. Recommendations for Next Generation Dataset and Testbed Design

To support scalable and trustworthy IDS, future datasets should:

- Combine multi-source data (telemetry, logs, flows) as in TON-IoT [160].
- Embed zero-day and adversarial attack simulations.
- Support low-latency formats (e.g., for TinyML or real-time IDS).
- Ensure privacy compliance and include federated learning annotations [84,161].

## 7. Robustness and Generalization of IoT Intrusion Detection Systems

While many intrusion detection systems report high detection accuracy on benchmark datasets, their performance often degrades when deployed in real-world environments. IoT networks are highly heterogeneous and dynamic, consisting of diverse devices, protocols, and traffic patterns. As a result, evaluating the robustness and generalization ability of IDS models is essential for ensuring reliable deployment. Several recent studies have highlighted that machine learning-based IDS models may achieve excellent performance on specific datasets but often fail to generalize to unseen environments due to dataset bias and limited training diversity. Therefore, robustness evaluation has become a critical requirement for the practical deployment of intelligent intrusion detection systems in IoT infrastructures [129,137,169].

### 7.1. Cross-Dataset Evaluation and Generalization

Most IDS models are evaluated on a single dataset, which may not capture the full diversity of real-world network environments [154,157]. Cross-dataset evaluation involves training a model on one dataset and testing it on another to measure its ability to generalize across different traffic patterns and attack behaviors. Several studies have emphasized that relying solely on a single benchmark dataset can lead to overfitting and unrealistic performance expectations.

Recent research in machine learning-based IoT security has shown that models trained on commonly used datasets such as UNSW-NB15, CICIDS, or Bot-IoT may experience a significant drop in detection accuracy when tested on different datasets due to variations in traffic distribution and attack characteristics. Consequently, cross-dataset validation has been recommended as a reliable strategy to assess the robustness and transferability of IDS models in heterogeneous IoT environments [32,160,161].

### 7.2. Adversarial Robustness and Evasion Defense

Deep learning-based IDS models are vulnerable to adversarial attacks in which carefully crafted inputs manipulate the detection outcome [6,96]. Attackers may inject malicious traffic patterns designed to evade detection or poison training data during the learning process. Recent studies highlight that adversarial perturbations can significantly degrade the performance of machine learning-based intrusion detection systems.

To mitigate these threats, several techniques have been proposed, including adversarial training, robust feature learning, and GAN-based data augmentation [12,106,138,142]. Additionally, XAI techniques have been explored to enhance the transparency and interpretability of IDS decisions, thereby enabling security analysts to understand model behavior better and detect potential adversarial manipulation.

Robust IDS deployment must also account for practical constraints, including device heterogeneity, limited computational resources, and dynamic network conditions. Many IoT devices have limited memory and processing capabilities, making it difficult to deploy complex deep learning models directly on edge nodes. Therefore, lightweight IDS models and distributed architectures have been proposed to balance detection performance and computational efficiency.

Edge computing and federated learning have emerged as promising solutions for improving robustness and scalability in IoT intrusion detection. Edge-based IDS frameworks enable real-time traffic analysis with reduced latency. At the same time, federated learning allows collaborative model training without sharing raw data, thereby preserving privacy and enhancing distributed detection capabilities.

Furthermore, hybrid architectures that integrate edge, fog, and cloud layers can improve resilience by distributing detection tasks across multiple levels of the IoT infrastructure [139,140]. Such hierarchical IDS designs enhance scalability and provide improved adaptability to dynamic network conditions.

Overall, robustness evaluation is essential for ensuring that IDS solutions remain effective across diverse deployment scenarios and evolving threat landscapes. Future research should focus on developing adaptive and resilient IDS frameworks capable of handling adversarial attacks, heterogeneous network environments, and continuously evolving cyber threats [73,95,149,169].

## 8. Open Challenges and Future Directions

**Figure 8:**
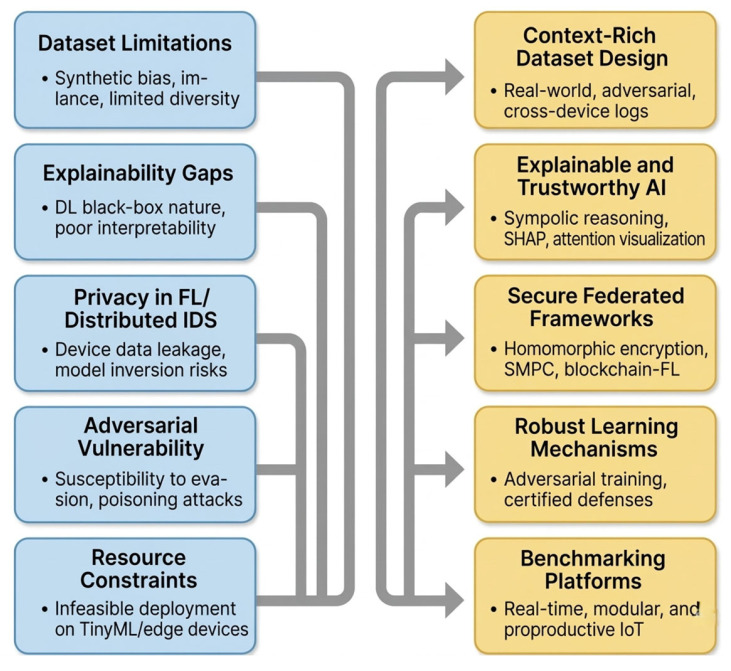
Open Challenges and Emerging Opportunities in AI-driven IoT IDS.

While IoT IDS has advanced significantly, many challenges still prevent widespread, long-term deployment [26]. These challenges span technical, usability, ethical, and scalability aspects. Addressing them requires a balanced approach, ensuring accuracy while maintaining interpretability, efficiency while preserving security, and innovation while complying with regulations [87,127]. Figure 8 illustrates how current IoT security challenges, such as Dataset Limitations and Resource Constraints, are being addressed by emerging solutions. It demonstrates a direct mapping from these problems to advanced methods such as Secure Federated Frameworks and Benchmarking Platforms.

### 8.1. The Accuracy-Interpretability Trade-Off

A major issue in IDS design lies in choosing between how accurate models are and how easy they are to understand. Deep learning and ensemble models, including CNNs, LSTMs, and Random Forests, often surpass conventional models in identifying complex attack patterns. But because they act as black boxes, it is hard to understand and trust them in sensitive domains like industrial IoT or healthcare systems [112,124,125]. On the other hand, simpler models like Decision Trees [153] or Naïve Bayes are easier to understand, but they might not work as well against more complex or hidden attacks. Regulatory requirements increasingly demand traceable and explainable AI models [57,119]. Emerging XAI tools, such as SHAP and LIME, offer some assistance, but determining how to integrate them effectively into lightweight IDS for edge devices with limited resources remains an open research area. Looking ahead, QML presents a theoretical paradigm shift. QML has the potential to process vast amounts of complex, multidimensional IoT traffic at unprecedented speeds, offering a future defense mechanism against quantum-level encryption-breaking attacks.

### 8.2. Practical Constraints: Resource, Realism, and Generalization

Most IoT devices have limited CPU power, memory, and energy, making them inherently resource-constrained. Deploying complex detection models, such as deep neural networks, on these devices is very challenging. Efforts like TinyML and model compression (pruning, quantization) show promise, but moving from cloud-trained models to edge devices often causes noticeable performance drops [14,107]. Finding the right balance between lightweight model design and strong threat detection remains a major challenge [15]. At the same time, the quality and realism of datasets remain important issues. Old datasets such as KDDCUP99 and BoT-IoT contain traffic that is either outdated or unrealistic, and even newer datasets such as CICIDS2017 are known for class imbalance [155,164]. Because of this, IDS models often do not work well in a wide range of real-world situations that are noisy or different. Privacy barriers also limit access to labeled real-world data. Research is exploring the use of GANs, transfer learning, and federated learning to generate synthetic datasets and bridge these gaps [12,102,138].

### 8.3. Emerging Vulnerabilities and Ethical Governance

IDS models themselves are becoming targets of attack. Adversarial examples can mislead models, while data poisoning threatens federated learning [96,117]. Beyond these risks, regulatory frameworks such as the EU AI Act enforce transparency, fairness, and accountability in AI systems. IoT IDS must respect privacy rights, avoid algorithmic bias, and ensure compliance across borders [57]. Responsible governance is, therefore, as important as technical innovation.

### 8.4. Toward Unified IDS Frameworks Across Domains

Today’s IDS solutions are often domain-specific, designed for contexts like IIoT, smart homes, or VANETs. This fragmentation reduces interoperability and raises costs. Researchers are now pursuing unified, modular IDS frameworks that adapt to different domains using transfer learning and meta-learning [102,161]. Standardized interfaces, datasets, and evaluation protocols will be key to enabling this shift toward more universal, scalable security solutions [92,171]. Table 16 provides a concise summary of these challenges and highlights opportunities for future IoT IDS research.

## 9. Conclusions

This review has provided a comprehensive and systematic analysis of Intrusion Detection Systems (IDS) tailored for IoT, with a focus on developments from 2021 to early 2026. We thoroughly examined the entire range of detection techniques, from basic signature- and anomaly-based systems to the most advanced AI-driven methods. We also critically analyzed various IDS architectures, explained IoT-specific attack taxonomies, detailed the evaluation of benchmark datasets and validation strategies, and mapped the evolving research landscape. Our principal contributions include the formulation of a structured taxonomy of IDS methodologies, the comparative analysis of supervised, unsupervised, deep, and hybrid models, and a comprehensive assessment of their performance within the inherent constraints of the IoT. We emphasized how recent advancements address significant issues in scalability, precision, and interpretability. The insights derived from this comprehensive review are integrated into curated tables and figures that provide a consolidated overview of findings across diverse datasets, methodologies, and architectures.

Several major challenges still face the field. We need to create more realistic datasets and find ways to deploy powerful AI models on small, low-power devices. It is also critical to develop robust defenses against sophisticated adversarial attacks, establish ethical guidelines for AI use, and develop mechanisms to govern AI ethically. Tackling these challenges requires cross-disciplinary collaboration spanning cybersecurity, machine learning, and embedded systems engineering. To this end, we advocate for next-generation IDS frameworks that are intelligent, adaptive, resource-aware, interoperable, and aligned with principles of explainability, privacy, and ethical AI. As IoT ecosystems expand and grow in complexity, future IDS solutions must remain scalable, interpretable, and resilient, ensuring they are not only effective today but also future-proof for tomorrow’s security demands.

## Figures and Tables

**Table 1:** Comparison with Recent IDS Surveys in IoT.

Ref.	Focus of Survey	AI Techniques	IDS Architectures	Deployment Strategies	Datasets & Validation	Emerging Techniques (XAI, FL, TinyML, LLMs)	Full-Scope Coverage
[8]	ML/DL in IoT security	✓	×	×	Limited	×	×
[6]	ML techniques for IoT Cyberattacks	✓ (ML/DL)	×	×	×	×	×
[9,10]	DL for IoT Zero-Day Threat Detection	✓ (DL)	×	×	Limited	×	×
[11]	Edge Computing and ML for IoT IDS	✓ (ML)	×	✓ (Edge-based)	✓	×	×
[12,13]	IDS Using GANs	✓ (GAN)	×	×	✓	✓ (GAN only)	×
[14,15]	Lightweight/TinyML IDS approaches	✓ (TinyML, ML)	×	✓ (TinyML focus)	×	✓ (TinyML)	×
[16,17]	XAI in intrusion detection systems	✓ (XAI, DL)	Partial	×	×	✓ (XAI)	×
[18]	Edge/Fog-based IDS architectures	✓ (DL/ML)	✓ (Edge-only)	✓	Partial	✓ (TinyML)	×
[19]	ML for IoT Security	✓	Partial	×	✓	×	×
[20]	LLM/Transformer	✓ LLMs and Transformer-IDS	Partial	✓	Partial	✓ (LLMs, Transformers)	×
[21]	Explainable DL IDS for IoT	✓ (DL, XAI)	Partial	×	✓	✓ (XAI only)	×
[22]	Comprehensive survey on DL-based IDS	✓ (DL)	Partial	Limited	✓	×	×
[23]	AI IDS training and deployment strategies	✓ (ML, DL, FL)	✓ (Edge, Fog, Cloud)	✓ (Edge, Cloud)	✓	(FL, Edge)	Partial
[24]	Metaheuristic and ML-driven IDS	✓ (ML, Metaheuristics)	Partial	×	Partial	✓ (Optimization)	×
[25]	ML/DL NIDS techniques for IoT	✓ (ML, DL)	Partial	✓	✓	Limited	×
[26]	Adaptive and Lightweight IDS	✓ (ML, DL)	Partial	✓ (Edge)	Partial	✓ (Incremental Learning, TinyML)	×
[27]	Quantum Machine Learning for IDS	✓ (QML)	×	×	Partial	✓ (QML Only)	×
	**This Review** **(Proposed)**	End-to-End IDS in IoT	✓ (DL, ML, Hybrid)	✓ (All: Centralized, Edge, Fog, Hybrid)	✓ (FL, Cloud, Edge TinyML)	✓ (10+ datasets, metrics)	✓ All Techniques including (XAI, FL, TinyML, GANs, LLMs, Transformer, LM, QML)

**Table 2:** Comparative Analysis of IDS Deployment Architectures in IoT Environments.

Architecture	Processing Location	Latency	Scalability	Privacy	Typical Techniques
Centralized	Cloud Server	High	Medium	Low	DL, LLMs, Ensembles
Distributed	Multiple Nodes	Medium	High	Medium	Federated Learning, DFL
Edge-Based	IoT Devices	Very Low	Medium	High	TinyML, Lightweight CNNs
Fog-Based	Fog Gateways	Low	High	Medium	XAI, Neurosymbolic Models
Hybrid	Edge + Fog + Cloud	Balanced	Very High	High	Multi-layer IDS, Split Learning

**Table 3:** Key DL Architectures and Their Applications.

Model	Key Strengths	Limitations	IoT Use Cases	Ref
FCNN,	Simple, fast for static data	Prone to overfitting	Smart homes, static sensor anomaly	[49,50]
RNN (LSTM/GRU)	Temporal modeling, sequence awareness	High training time, vanishing gradients	Wearable health, smart grid logs	[51]
CNN	Spatial feature extraction	Needs structured inputs	Traffic flow analysis, ICS	[52]
GAN	Synthetic data generation	Training instability	Data augmentation in constrained IoT	[53]
Auto-encoder	Compression, anomaly reconstruction	Sensitive to noise and reconstruction tuning	Health IoT, unsupervised anomaly detection	[54]

**Table 4:** Deployment Strategies and Their Tradeoffs.

Strategy	Strengths	Limitations	Best-Fit Techniques	Domain Examples
On-Device	Real-time detection,	Limited to very small	TinyML, rule-based	Smart home, wearables
	strong privacy	models	models	
Gateway	Supports mid-size	May become	Fuzzy logic, lightweight	Smart meters, HVAC
	models, low latency	performance bottleneck	CNN	systems
Fog/Edge	Local processing, reduces	Maintenance overhead,	Autoencoders, LSTM	Industrial IoT, smart
	bandwidth	hardware costs		factories
Cloud	Scalable analytics,	Higher latency, privacy	GANs, LLMs	Smart city, intelligent
	powerful storage	risks		transport
Federated	Preserves privacy,	Model sync issues,	Federated Learning,	Healthcare, distributed
	decentralized data	poisoning risks	neuro-symbolic AI	sensors

**Table 5:** IoT Layer-wise Attack Taxonomy, Design Implications, and Emerging IDS Responses.

IoT Layer	Example Attacks	IDS Design Implication	Reported Performance	References
Perception	Node tampering, fake node injection, replay, jamming	Lightweight on-device anomaly detection, RF fingerprinting, hardware authentication (PUFs)	~90–94% accuracy on lightweight edge IDS; real-time anomaly detection	[71,77,78]
Network	Sinkhole, Sybil, forwarding, wormhole, RPL spoofing, DoS	Graph/topology-aware IDS, flow-based edge/fog detection, Federated edge learning	95–97% F1-score on TON_IoT; protocol-resilient	[79,80,84,85]
Application	Malware, phishing, API abuse, code injection	Application-layer behavioral modeling, log analysis, XAI-based alert explanation	97–99% accuracy in malware/phishing detection using DL models	[88,89,90,93]
Advanced Threats	Adversarial ML, insider, privacy leakage, multi-stage attacks	Adversarial training, temporal/Transformer-based sequence modeling, neuro-symbolic logic	91–97% detection rate for adversarial and multi-stage attacks	[55,95,97]

**Table 6:** Representative Studies: Conceptual Contributions and Reported Empirical Performance of Emerging IoT IDS.

Technique/Ref	Addressed Limitation	Key Contribution	Dataset & Validation Setting	Reported Performance & Resources
Federated Learning (FL) [98,99,100,101,102]	Centralized training risks, data imbalance, and non-IID data.	FedMSE and Chimp-optimized FL improve resilience, reduce data leakage, and minimize model divergence.	N-BaIoT, Smart environments (Holdout/Client-split)	95.6% to 97.3% accuracy. Reduces bandwidth but faces synchronization lag.
FL + Transformers [103]	Long-range pattern detection, privacy.	Enables advanced contextual sequence modeling across distributed nodes.	N-BaIoT, UNSW-NB15, CICIoT2023	99%+ accuracy/F1/precision. High computational cost.
FL + Blockchain [104]	Trust and tampering in FL Aggregation.	Blockchain-secured FL aggregation ensures tamper resistance.	Distributed IoT IDS (Network split)	97.3% accuracy; reduces communication cost by 41%.
FL + Explainable AI [105]	Black-box ML models, lack of interpretability.	Integrates SHAP with FL to provide transparent, interpretable alerts.	CICIoT2023 (Holdout)	~88% accuracy. Explanations add computational overhead.
GANs for IDS [12,106]	Zero-day attacks, severe class imbalance.	GAN-LSTM generates synthetic minority attacks to train highly resilient models.	Malware/polymorphic setting (Benchmark split)	98.2% accuracy. Significantly improves rare attack detection.
TinyML for Edge IDS [14,107,108]	Resource constraints, high latency, lack of local learning.	Hardware-aware TinyML enables direct local inference, reducing cloud dependency.	Edge IDS setting (Device-level testing)	99.50% accuracy, 99.45% F1, 4.5 s computation time. Extremely low memory footprint.
Transformers/LLMs [56,109]	Weak natural language reasoning for logs, RNN limits.	IoT-BERT and LLMs improve semantic understanding of telemetry and log data.	Telemetry/Log data (Sequence-based evaluation)	Superior long-dependency anomaly detection and interpretable alerts. High compute requirement.
Neurosymbolic and Responsible AI [110,111,112]	Poor policy alignment, ethical bias, lack of explanations.	Combines neural and symbolic graphs (SymbolNet-ID) for fair, auditable governance.	IoT security policy-aware settings (Conceptual/Prototype)	Multi-layer explainability and policy compliance. Added reasoning overhead.

**Table 7:** Responsible and Explainable AI Requirements across the IDS Design Lifecycle.

IDS Design Phase	Responsible/XAI Requirement	References
Dataset Collection and Preprocessing	Bias control, privacy preservation, class balancing, anonymization.	[9,130,131]
Model Training and Validation	Fairness checks, adversarial robustness, accuracy-explainability trade-offs.	[21,94,96]
Deployment and Alert Generation	SHAP/LIME integration, explanation latency, and resource constraints (TinyML).	[14,21,120,132]
Federated and Update Cycles	Secure aggregation, poisoned-update detection, and privacy-preservation.	[55,114,117]

**Table 8:** Selected Applications of GAN-Based IDS in IoT Environments.

Ref	GAN Variant	Target Threat/Use Case	Performance/Highlight
[139]	FederatedcGAN (WGAN-GP)	Detects zero-day and adversarial attacks in IIoT	Achieved ~10% higher accuracy than FedID
[137]	SAPGAN (Self-Attention Progressive GAN)	Detects IoT attacks (DDoS, RTSP brute force, camera flood)	improves accuracy by up to ~27% and reduces computation time
[142]	WGAN-AE (Hybrid Wasserstein GAN + Autoencoder)	Detects IoT attacks with high accuracy	accuracy (~97%), PR-AUC up to 99.8%, low memory (~60 kB)

**Table 9:** Applications and Performance of LLM and Transformer-Based IDS Models.

Study/Model	Focus	Performance Outcome
[146]—LTM-based IDS (BERT, DistilBERT, RoBERTa)	IoT attack classification	achieves low loss and strong generalization, enabling real-time detection
[144]—BERT-GRU IDS	Network traffic as text for intrusion detection	improves accuracy and detection of complex attack patterns
[145]—BT-TPF (Distilled Transformer-ViT + Poolformer)	Lightweight IoT intrusion detection	achieves > 99% accuracy with ~90% parameter reduction

**Table 10:** Neuro-symbolic vs. Neural vs. Symbolic IDS Models.

Model Type	Strengths	Limitations
Symbolic-only IDS	Time-series anomaly detection	Inflexible to novel attacks, hardcoded logic
Neural-only IDS	Good at detecting novel patterns, data-driven	Opaque decisions, needs large data, prone to bias
Neuro-symbolic IDS	Combines learning and logic, explainable alerts	Complex to build, needs both data + expert Knowledge

**Table 11:** Responsible AI Principles in IoT IDS.

Principle	Implementation in IDS	Challenges
Fairness	Dataset balancing, demographic parity auditing	Dynamic IoT context, hidden bias
Explainability	SHAP, LIME, rule-based outputs, visual dashboards	Added compute load, limited edge interpretability
Accountability	Logging, traceability, human In the loop alerts	Policy enforcement, legal ambiguity
Privacy	Federated Learning, SMPC, local inference, differential privacy	Trade-offs with detection accuracy

**Table 12:** Traditional vs. Emerging IDS Techniques.

Technique	Pros	Cons
Signature-Based IDS	Efficient at detecting known attacks, with low resource consumption	Cannot detect novel attacks, requires constant updates
Anomaly-Based IDS	Detects unknown threats, adaptable	Added compute load, limited edge interpretability
XAI	Improved interpretability, higher accountability	Additional computational overhead
Federated Learning	Privacy-preserving, scalable	Vulnerable to model poisoning, Communication overhead
TinyML	Low resource usage, real-time detection	Limited model complexity, deployment challenges
GANs	Enhanced training data, improved robustness	Training instability, computationally intensive
SMPC	Privacy-preserving enables collaboration	High computational cost, limited scalability

**Table 13:** Adaptive IoT IDS: Challenges and Solutions.

Adaptability Issue	Suitable Approach	Remaining Risk
Concept drift [147,148]	Drift-aware incremental learning.	False adaptation if drift is missed.
Zero-day attacks [10,73]	Continual learning with new data.	Label scarcity; delayed ground truth.
Non-IID traffic [62,116,148]	Incremental federated learning.	Client drift and synchronization lag.
Forgetting attacks [148]	Replay memory, knowledge Distillation.	High memory and computing overhead.
Poisoned updates [104,115]	Secure aggregation, blockchain Validation.	Added communication costs.

**Table 14:** Comprehensive Evaluation of Dataset Realism, Limitations, and Generalization Risks in IoT IDS.

Dataset Category and Properties	Data Type/Size	Key Characteristics and Attack Types	Inherent Limitations and Validation Best Practices
Legacy Benchmarks DARPA 98, KDDCUP 99, NSL-KDD [153,154,155]	Type: Non-IoT/ Tabular and TCP Dump Size: Medium to Very Large	Attack Types: DoS, R2L, U2R, Basic/Derived attacks. Strengths: Structured benchmarks, widely cited, improved KDD versions.	Limitations: Synthetic data, completely Lacks IoT traffic, redundant, and outdated threats. Risk/Practice: Extreme risk. Models will fail in modern IoT. Discontinue for active evaluation; use as historical baselines.
General NIDS (Enterprise and Flow) ISCX 2012, ADFA, UNSW-NB15, CICIDS2017, CAIDA [41,156,158,168]	Type: Non-to-Partial IoT/PCAP, Flow, Syscalls Size: Moderate to Very Large	Attack Types: HTTP/SSH/FTP, Zero-day exploits, 9-classes, DDoS, Multi-class. Strengths: Detailed labeling, real traffic scale, DL/FL-compatible.	Limitations: Enterprise-focused, no device-specific labeling, not tailored for IoT, high resource demand. Risk/Practice: High risk for IoT constraints. Use only as supplementary data for general network anomaly detection.
First-Gen IoT BoT-IoT (2018) [159,165]	Type: IoT-Centric/PCAP + Flow Size: Very Large	Attack Types: 4+ attacks (Botnet DoS/DDoS, Reconnaissance, Data Exfiltration). Strengths: Specifically designed For early IoT threat detection.	Limitations: Relies on simulated traffic generation and suffers from severe class imbalance. Risk/Practice: High risk. Accuracy metrics are easily inflated. Enforce imbalance-aware metrics (macro-F1) and stratified cross-validation.
Multimodal IoT TON-IoT (2020) [160,161]	Type: IoT and IIoT/Telemetry + Logs Size: Very Large	Attack Types: 20+ attacks (Ransomware, MITM, Password Cracking, etc.). Strengths: Provides rich, Multi-modal IoT data sources.	Limitations: Requires heavy preprocessing to adequately fuse network flows, telemetry, and OS logs. Risk/Practice: Moderate risk. Preprocessing variability leads to inconsistent benchmarking. Ideal for evaluating cross-environment models.
Advanced IoT CICIoT2023 (2023) [162,163,164]	Type: Modern IoT PCAP + NetFlows Size: Large	Attack Types: Modern + adversarial (Mirai/BashLite, RPL/ARP Spoofing, Replay). Strengths: Offers the latest, highly realistic adversarial Representation for IoT.	Limitations: Memory-intensive with a massive memory footprint; computationally expensive to train for edge deployment. Risk/Practice: Low detection risk but high deployment risk. Mandate time-aware (temporal) and cross-device splits.

**Table 15:** Framework for Robust IoT IDS Validation and Evaluation.

Validation Protocol	Purpose (Addressing Evaluation Flaws)	Recommended Action and Metrics
Cross-Dataset [41,154,163]	Tests generalization; prevents dataset-specific overfitting.	Train on one dataset, test on another (e.g., BoT-IoT → TON_IoT).
Time-Aware [23,147,148]	Evaluates robustness against concept drift and evolving attacks.	Use strict chronological train/test splits; avoid random holdout.
Device-Level [25,41]	Checks reliability across heterogeneous, unseen IoT nodes.	Apply leave-one-device-out testing.
Stratified/Macro-F1 [9,130,131]	Prevents misleading accuracy in highly imbalanced datasets.	Use stratified splits; strictly report Macro-F1 and per-class F1.
FPR and AUC-ROC [34,160]	High FPR renders an IDS unusable due to alert fatigue.	Always report FPR with Precision/Recall. Use PR-AUC for rare attacks.
Resource Metrics [68,69,132]	Ensures edge/TinyML deployment is practically feasible.	Report inference latency, memory footprint, and CPU/energy overhead.

**Table 16:** Summary of Open Challenges and Emerging Opportunities in IoT IDS.

Challenge	Description	Why It Matters	Opportunities/Solutions
Interpretability vs. Accuracy	High-performing models are often black-box [112,124,125]	Reduces trust, hinders compliance in critical domains	Use of XAI (e.g., SHAP, LIME); neuro-symbolic AI
Power and Memory Constraints	IoT nodes have limited computational and energy resources [14,107]	Limits the deployment of complex models	TinyML, model compression, edge fog hybrid strategies
Dataset Realism and Generalization	Existing datasets may be synthetic, outdated, or imbalanced	Models fail to generalize to real-world traffic [164]	GAN-generated datasets, data augmentation, federated learning [169]
Security of ML Models	Models are vulnerable to adversarial and poisoning attacks [96,106]	IDS itself becomes a security liability	Adversarial training, secure FL aggregation, model watermarking
Cross-Device Synchronization	Inconsistent clocks/formats across IoT nodes	Hinders distributed model performance	Robust FL protocols, scalable architectures, time-agnostic modeling
Regulatory and Ethical Concerns	Transparency and fairness are mandated by GDPR/AI laws [57]	Risk of legal non-compliance, bias	Responsible AI toolkits, auditable IDS, and explainable decision making
Fragmented Frameworks Across Domains	Domain-specific IDS hinders interoperability [102,161]	Increases cost and system complexity	Unified modular frameworks, domain adaptation, transfer learning

## Data Availability

No new data or code were generated in this study. All data supporting this review are from previously published sources, which are appropriately cited.

## Abbreviation

List of Acronyms and Abbreviations.

**Acronym**	**Full Form**
IDS	Intrusion Detection System
AIDS	Anomaly-based Intrusion Detection System
RNN	Recurrent Neural Network
CNN	Convolutional Neural Network
FL	Federated Learning
DFL	Distributed Federated Learning
DL	Deep Learning
DoS/DDoS	Denial of Service/Distributed Denial of Service
DT	Decision Tree
GAN	Generative Adversarial Network
HIDS	Host-based Intrusion Detection System
LLM	Large Language Model
IIoT	Industrial Internet of Things
IoT	Internet of Things
KNN	K-Nearest Neighbors
LSTM	Long Short-Term Memory
ML	Machine Learning
NIDS	Network-based Intrusion Detection System
RPL	Routing Protocol for Low-Power and Lossy Networks
SIDS	Signature-based Intrusion Detection System
SMPC	Secure Multi-Party Computation
SVM	Support Vector Machine
ICS	Industrial Control Systems
RL	Reinforcement Learning
GNN	Graph Neural Network
AUC	Area Under the Curve
FPR	False Positive Rate
QML	Quantum Machine Learning
PR-AUC	Precision–Recall Area Under the Curve
XAI	Explainable Artificial Intelligence
TinyML	Tiny Machine Learning

## References

[1] Ahmad Z., Shahid Khan A., Shiang C., Ahmad F. (2021). Network intrusion detection system: A systematic study of machine learning and deep learning approaches. Trans. Emerg. Telecommun. Technol..

[2] Alem S., Espes D., Nana L., Martin E., De Lamotte F. (2023). A novel bi-anomaly-based intrusion detection system approach for industry 4.0. Future Gener. Comput. Syst..

[3] Aydin B., Aydin H., Gormus S. (2025). Intrusion detection systems in IoT: A detailed review of threat categories, detection strategies, and future technologies. J. Inf. Secur. Appl..

[4] Benameur R., Dahane A., Souihi S., Mellouk A. A Novel Federated Learning Based Intrusion Detection System for IoT Networks. Proceedings of the ICC 2024—IEEE International Conference on Communications.

[5] Berhili M., Chaieb O., Benabdellah M. (2024). Intrusion Detection Systems in IoT Based on Machine Learning: A state of the art. Procedia Comput. Sci..

[6] Bout E., Loscri V., Gallais A. (2022). How Machine Learning Changes the Nature of Cyberattacks on IoT Networks: A Survey. IEEE Commun. Surv. Tutor..

[7] Elrawy M.F., Awad A.I., Hamed H.F.A. (2018). Intrusion detection systems for IoT-based smart environments: A survey. J. Cloud Comput..

[8] Ferrag M.A., Shu L., Friha O., Yang X. (2022). Cyber Security Intrusion Detection for Agriculture 4.0: Machine Learning-Based Solutions, Datasets, and Future Directions. IEEE/CAA J. Autom. Sin..

[9] Ferrag M.A., Maglaras L., Moschoyiannis S., Janicke H. (2020). Deep learning for cyber security intrusion detection: Approaches, datasets, and comparative study. J. Inf. Secur. Appl..

[10] Guo Y. (2023). A review of Machine Learning-based zero-day attack detection: Challenges and future directions. Comput. Commun..

[11] Gyamfi E., Jurcut A. (2022). Intrusion Detection in Internet of Things Systems: A Review on Design Approaches Leveraging Multi-Access Edge Computing, Machine Learning, and Datasets. Sensors.

[12] Dunmore A., Jang-Jaccard J., Sabrina F., Kwak J. (2023). A Comprehensive Survey of Generative Adversarial Networks (GANs) in Cybersecurity Intrusion Detection. IEEE Access.

[13] Alauthman M., Aslam N., Al-Qerem A., Aldweesh A., Sureephong P. (2026). Generative Adversarial Networks for Intrusion Detection Systems: A Comprehensive Survey of Applications, Challenges, and Research Directions. Arab. J. Sci. Eng..

[14] Amuthadevi C., Venkatesan R., Mythily M., Canessane R.A. (2025). TinyML-based intrusion detection systems for sustainable and energy-constrained IoT devices. Results Eng..

[15] Alwaisi Z., Kumar T., Harjula E., Soderi S. (2024). Securing constrained IoT systems: A lightweight machine learning approach for anomaly detection and prevention. Internet Things.

[16] Neupane S., Ables J., Anderson W., Mittal S., Rahimi S., Banicescu I. (2022). Explainable Intrusion Detection Systems (X-IDS): A Survey of Current Methods, Challenges, and Opportunities. IEEE Access.

[17] Khan N., Ahmad K., Al Tamimi A., Alani M.M., Bermak A., Khalil I. (2025). Explainable AI-Based Intrusion Detection Systems for Industry 5.0 and Adversarial XAI: A Systematic Review. Information.

[18] Chaturvedi P., Ahmad S., Mewada A. (2026). A Comprehensive Survey on Fog Computing: Architectures, Techniques, Challenges, and Future Directions. Arch. Comput. Methods Eng..

[19] Hozouri A., Mirzaei A., Effatparvar M. (2025). A comprehensive survey on intrusion detection systems with advances in machine learning, deep learning and emerging cybersecurity challenges. Discov. Artif. Intell..

[20] Jaffal N.O., Alkhanafseh M., Mohaisen D. (2025). Large Language Models in Cybersecurity: A Survey of Applications, Vulnerabilities, and Defense Techniques. AI.

[21] Ogunseyi T.B., Thiyagarajan G., He H., Bist V., Du Z. (2026). Performance Analysis of Explainable Deep Learning-Based Intrusion Detection Systems for IoT Networks: A Systematic Review. Sensors.

[22] Al-Haija Q.A., Droos A. (2025). A comprehensive survey on deep learning-based intrusion detection systems in Internet of Things (IoT). Expert Syst..

[23] Mallidi S.K.R., Ramisetty R.R. (2025). Advancements in training and deployment strategies for AI-based intrusion detection systems in IoT: A systematic literature review. Discov. Internet Things.

[24] Ahsan M.S., Islam S., Shatabda S. (2025). A systematic review of metaheuristics-based and machine learning-driven intrusion detection systems in IoT. Swarm Evol. Comput..

[25] Walling S., Lodh S. (2025). An Extensive Review of Machine Learning and Deep Learning techniques on network intrusion detection for IoT. Trans. Emerg. Telecommun. Technol..

[26] Suzan S., El Barachi M., Li N. (2026). Intrusion Detection on the Internet of Things: A Comprehensive Review and Gap Analysis Toward Real-Time, Lightweight, Adaptive, and Autonomous Security. IoT.

[27] Chaudhary D., Rajasegarar S., Pokhrel S.R. (2025). Towards Adapting Federated & Quantum Machine Learning for Network Intrusion Detection: A Survey. arXiv.

[28] Diana L., Dini P., Paolini D. (2025). Overview on Intrusion Detection Systems for Computers Networking Security. Computers.

[29] Díaz-Verdejo J., Muñoz-Calle J., Alonso A.E., Alonso R.E., Madinabeitia G. (2022). On the Detection Capabilities of Signature-Based Intrusion Detection Systems in the Context of Web Attacks. Appl. Sci..

[30] Khraisat A., Gondal I., Vamplew P., Kamruzzaman J. (2019). Survey of intrusion detection systems: Techniques, datasets and challenges. Cybersecurity.

[31] Khraisat A., Alazab A. (2021). A critical review of intrusion detection systems in the internet of things: Techniques, deployment strategy, validation strategy, attacks, public datasets and challenges. Cybersecurity.

[32] Kumar L.K.S., Nethi S.R., Uyyala R., Vurubindi P., Narahari S.C., Das A.K. (2026). Anomaly-based intrusion detection on benchmark datasets for network security: A comprehensive evaluation. Sci. Rep..

[33] Khacha A., Aliouat Z., Harbi Y., Gherbi C., Saadouni R., Harous S. (2024). Landscape of learning techniques for intrusion detection system in IoT: A systematic literature review. Comput. Electr. Eng..

[34] Faruqui N., Yousuf M.A., Whaiduzzaman M., Azad A.K.M., Alyami S.A., Liò P., Kabir M.A., Moni M.A. (2023). SafetyMed: A Novel IoMT Intrusion Detection System Using CNN-LSTM Hybridization. Electronics.

[35] Hnamte V., Nhung-Nguyen H., Hussain J., Hwa-Kim Y. (2023). A Novel Two-Stage Deep Learning Model for Network Intrusion Detection: LSTM-AE. IEEE Access.

[36] Afraji D.M.A.A., Lloret J., Peñalver L. (2025). An Integrated Hybrid Deep Learning Framework for Intrusion Detection in IoT and IIoT Networks Using CNN-LSTM-GRU Architecture. Computers.

[37] Nguyen T., Janapa Reddi V. (2021). Deep Reinforcement Learning for Cyber Security. IEEE Trans. Neural Netw. Learn. Syst..

[38] Samita (2024). A Review on Intrusion Detection System for IoT based Systems. SN Comput. Sci..

[39] Sharma B., Sharma L., Lal C., Roy S. (2024). Explainable artificial intelligence for intrusion detection in IoT networks: A deep learning based approach. Expert Syst. Appl..

[40] Ogunbadejo M., Alade O. (2025). Machine Learning Methods for Intrusion Detection: A Comprehensive Survey. Int. J. Sci. Res. Manag..

[41] Bertoli G.C., Junior L.A.P., Verri F.A.N., Santos A.L., Saotome O. (2021). Bridging the gap to real-world for network intrusion detection systems with data-centric approach. arXiv.

[42] Babu A., Bagubali A. (2025). Federated Learning With Sailfish-Optimized Ensemble Models for Anomaly Detection in IoT Edge Computing Environment. IEEE Access.

[43] Nguyen T.D., Alazab A., Khraisat A., Jan T. (2026). Feature reduction in federated learning for intrusion detection in IoT networks. Cybersecurity.

[44] Rey V., Sánchez Sánchez P.M., Huertas Celdrán A., Bovet G. (2022). Federated learning for malware detection in IoT devices. Comput. Netw..

[45] Neto E.C.P., Iqbal S., Buffett S., Sultana M., Taylor A. (2025). Deep learning for intrusion detection in emerging technologies: A comprehensive survey and new perspectives. Artif. Intell. Rev..

[46] Almuhanna R., Dardouri S. (2025). A deep learning/machine learning approach for anomaly based network intrusion detection. Front. Artif. Intell..

[47] Panneerselvam N., Krithiga S. (2022). A novel security framework for densely populated Internet of Things users in pervasive service access. Comput. Commun..

[48] Lee J., Park K.-H. (2019). GAN-Based Imbalanced Data Intrusion Detection System. Pers. Ubiquitous Comput..

[49] Almasabi A.M., Alkhodre A.B., Khemakhem M., Eassa F., Abi Sen A.A., Harbaoui A. (2025). Internet of Things-Based Anomaly Detection Hybrid Framework Simulation Integration of Deep Learning and Blockchain. Information.

[50] Fouad Y., Abdelaziz N.E., Elshewey A.M. (2024). IoT Traffic Parameter Classification based on Optimized BPSO for Enabling Green Wireless Networks. Eng. Technol. Appl. Sci. Res..

[51] Alayash W., Rahrouh M., Ibrahim A.A., Mohamed M.H., Ahmed S.T., Albarri M.H., Ahmed M.H. (2026). Assessing LSTM and GRU for Multi-Dataset Intrusion Detection in IoT Environments. Stat. Optim. Inf. Comput..

[52] Sadhwani S., Khan M.A.H., Muthalagu R., Pawar P.M., Suresh K. (2025). A hybrid BiLSTM-CNN approach for intrusion detection for IoT applications. Sci. Rep..

[53] Siam A.A., Alazab M., Awajan A., Faruqui N. (2025). A Comprehensive Review of AI’s Current Impact and Future Prospects in Cybersecurity. IEEE Access.

[54] Yao W., Hu L., Hou Y., Li X. (2023). A Lightweight Intelligent Network Intrusion Detection System Using One-Class Autoencoder and Ensemble Learning for IoT. Sensors.

[55] Vyas A., Lin P.C., Hwang R.H., Tripathi M. (2024). Privacy-Preserving Federated Learning for Intrusion Detection in IoT Environments: A Survey. IEEE Access.

[56] Tseng S.-M., Wang Y.-Q., Wang Y.-C. (2024). Multi-Class Intrusion Detection Based on Transformer for IoT Networks Using CIC-IoT-2023 Dataset. Future Internet.

[57] Kaur I., Sikka R. (2024). Towards Responsible AI in Cybersecurity: Current Trends, Ethical Considerations, and Best Practices. Natl. Res. J. Inf. Technol. Inf. Sci..

[58] Alabbadi A., Bajaber F. (2025). An Intrusion Detection System over the IoT Data Streams Using eXplainable Artificial Intelligence (XAI). Sensors.

[59] Rahman M.A., Asyhari A.T., Leong L.S., Satrya G.B., Tao M., Zolkipli M.F. (2020). Scalable machine learning-based intrusion detection system for IoT-enabled smart cities. Sustain. Cities Soc..

[60] Isong B., Kgote O., Abu-Mahfouz A. (2024). Insights into Modern Intrusion Detection Strategies for Internet of Things Ecosystems. Electronics.

[61] Alotaibi Y., Ilyas M. (2023). Ensemble-Learning Framework for Intrusion Detection to Enhance Internet of Things’ Devices Security. Sensors.

[62] Ali Khan M., Rais R.N.B., Khalid O., Deriche M. (2025). Comparative Analysis of Centralized and Federated Intrusion Detection in IoT-Enabled Cyber-Physical Systems Under Data and Label-Skew. IEEE Access.

[63] Aldaej A., Ullah I., Ahanger T.A., Atiquzzaman M. (2024). Ensemble technique of intrusion detection for IoT-edge platform. Sci. Rep..

[64] Qaddos A., Yaseen M.U., Al-Shamayleh A.S., Imran M., Akhunzada A., Alharthi S.Z. (2024). A novel intrusion detection framework for optimizing IoT security. Sci. Rep..

[65] Ponniah K.K., Retnaswamy B. (2023). A novel deep learning based intrusion detection system for the IoT-Cloud platform with blockchain and data encryption mechanisms. J. Intell. Fuzzy Syst..

[66] Alsulami A.A., Abu Al-Haija Q., Tayeb A., Alqahtani A. (2022). An Intrusion Detection and Classification System for IoT Traffic with Improved Data Engineering. Appl. Sci..

[67] Hizal S., Cavusoglu U., Akgun D. (2024). A novel deep learning-based intrusion detection system for IoT DDoS security. Internet Things.

[68] Panopio A.J.N., Abushahla H.A., Sajun A.R., Alawnah S., Aloul F., Zualkernan I. (2026). From Sensor to Server: Deployable Lightweight ML for IoT Intrusion Detection Across Network Layers. IEEE Internet Things J..

[69] N S.S., P P., Jain K., Krishnan P. (2026). Edge AI Bridge: A Micro-Layer Intrusion Detection Architecture for Smart-City IoT Networks. IoT.

[70] Jangra N., Rana R.P.S. Perception To Application Layer: A Critical Review On Vulnerability Assessment of Iot Device. Proceedings of the 2024 15th International Conference on Computing Communication and Networking Technologies (ICCCNT).

[71] Racherla S., Sripathi P., Faruqui N., Kabir M.A., Whaiduzzaman M., Shah S.A. (2024). Deep-IDS: A Real-Time Intrusion Detector for IoT Nodes Using Deep Learning. IEEE Access.

[72] Munshar H.H.A., Jemili F., Korbaa O., Alauthmaan M. (2026). Comprehensive analysis of intrusion detection systems for enhancing security in internet of things environments. Discov. Appl. Sci..

[73] Bas S., Kaya K., Ak E., Oguducu S.G. Adaptive Intrusion Detection for Evolving RPL IoT Attacks Using Incremental Learning. Proceedings of the 2026 IEEE 23rd Consumer Communications & Networking Conference (CCNC).

[74] Jader U.H., Kurda R., Muhamad S.R. (2026). Navigating Cyber Threats: The Role of Machine Learning and Deep Learning in Fifth-Generation Internet of Things Security. ARO-Sci. J. KOYA Univ..

[75] Rauf M.H., Usman M. (2025). Comprehensive Review of Challenges and Solutions for Physical Layer Security in IoT Networks. ICTACT J. Commun. Technol..

[76] Nasereddin M., Gelenbe E. (2025). A Survey of the Security of IoT Network Layers. TechRxiv.

[77] Amamra A., Nguyen V., Cheung A., Acosta S., Pham T.L. (2026). Wavelet-Based IoT Device Fingerprinting. Electronics.

[78] Feng P., Li B., Han B., Ma Y., Hu Y., Zhao R. (2026). A Scalable Group Authentication Protocol for IoT Based on PUF-Derived Bases in Inner Product Spaces. Cybersecurity.

[79] Li J., Wang Z. (2024). Sybil Attack Detection for Secure IoT-Based Smart Healthcare Environments. J. Inst. Eng. India Ser. B.

[80] Zhukabayeva T., Zholshiyeva L., Mardenov Y., Buja A., Khan S., Alnazzawi N. (2025). Real-Time Detection and Response to Wormhole and Sinkhole Attacks in Wireless Sensor Networks. Technologies.

[81] Abuagoub A. (2024). Security concerns with IoT routing: A review of attacks, countermeasures, and future prospects. Adv. Internet Things.

[82] Pham Le P.-H., Do Q.N., Dinh T.Q., Pham H.-T.-N., Nguyen L.V. (2026). A comparative security analysis of MQTT brokers against DoS attacks. J. Inf. Secur..

[83] Das R., Deka V., Devi R., Dey A., Sharma M., Taye G. (2026). Advancements in AI-Based Botnet Detection Techniques for IoT Networks: A Comprehensive Survey. Proceedings of the NIELIT’s International Conference on Communication, Electronics and Digital Technologies.

[84] Khraisat A., Alazab A., Singh S., Jan T., Gomez A.J. (2024). Survey on Federated Learning for Intrusion Detection System: Concept, Architectures, Aggregation Strategies, Challenges, and Future Directions. ACM Comput. Surv..

[85] Bilot T., Madhoun N.E., Agha K.A., Zouaoui A. (2023). Graph Neural Networks for Intrusion Detection: A Survey. IEEE Access.

[86] Singh S., Sharma M., Hossain S.A. (2024). Navigating the Threat Landscape of IoT: An Analysis of Attacks. Innovative Computing and Communication.

[87] Rawat M., Singal G. (2025). Surveying Technology Fusion in IoT Networks for IDS: Exploring Datasets, Tools, Challenges, and Research Prospects. ACM Trans. Intell. Syst. Technol..

[88] Garg H., Dave M. Securing IoT Devices and Securely Connecting the Dots Using REST API and Middleware. Proceedings of the 2019 4th International Conference on Internet of Things: Smart Innovation and Usages (IoT-SIU).

[89] Al-Shurbaji T., Anbar M., Manickam S., Hasbullah I.H., Alfriehat N., Alabsi B.A. (2025). Deep Learning-Based Intrusion Detection System for Detecting IoT Botnet Attacks: A Review. IEEE Access.

[90] Abbas S.G., Vaccari I., Hussain F., Zahid S., Fayyaz U.U., Shah G.A. (2021). Identifying and Mitigating Phishing Attack Threats in IoT Use Cases Using a Threat Modelling Approach. Sensors.

[91] Lightbody D., Ngo D.-M., Temko A., Murphy C.C., Popovici E. (2023). Attacks on IoT: Side-Channel Power Acquisition Framework for Intrusion Detection. Future Internet.

[92] Abdulkareem S.A., Foh C.H., Shojafar M., Carrez F., Moessner K. (2024). Network Intrusion Detection: An IoT and Non IoT-Related Survey. IEEE Access.

[93] Mohale V.Z., Obagbuwa I.C. (2025). Evaluating machine learning-based intrusion detection systems with explainable AI: Enhancing transparency and interpretability. Front. Comput. Sci..

[94] Hossain M.J., Alam K., Monir M.F., Hoque M.M., Ahmed T. (2025). Explainable AI Meets Synthetic Data: A Deep Learning framework for Detecting Network Intrusion in NextG Network Infrastructure. IEEE Access.

[95] Al Rawajbeh M., Maria Soosai A.J., Ramasamy L.K., Khan F. (2025). Trustworthy Adaptive AI for Real-Time Intrusion Detection in Industrial IoT Security. IoT.

[96] Velliyath S., Kalaivani D. Adversarial Challenges in AI-based Intrusion Detection Systems for Cloud Environments: A Comprehensive Review. Proceedings of the 2025 5th International Conference on Evolutionary Computing and Mobile Sustainable Networks (ICECMSN).

[97] Verkerken M., D’hooge L., Sudyana D., Lin Y.D., Wauters T., Volckaert B. (2023). A Novel Multi-Stage Approach for Hierarchical Intrusion Detection. IEEE Trans. Netw. Serv. Manag..

[98] Sha K., Yang T.A., Wei W., Davari S. (2020). A Survey of Edge Computing-Based Designs for IoT Security. Digit. Commun. Netw..

[99] Karunamurthy A., Vijayan K., Kshirsagar P.R., Tan K.T. (2025). An optimal federated learning-based intrusion detection for IoT environment. Sci. Rep..

[100] Khraisat A., Alazab A., Alazab M., Obeidat A., Singh S., Jan T. (2025). Federated learning for intrusion detection in IoT environments: A privacy-preserving strategy. Discov. Internet Things.

[101] Albanbay N., Tursynbek Y., Graffi K., Uskenbayeva R., Kalpeyeva Z., Abilkaiyr Z. (2025). Federated Learning-Based Intrusion Detection in IoT Networks: Performance Evaluation and Data Scaling Study. J. Sens. Actuator Netw..

[102] Nguyen V.T., Beuran R. (2025). FedMSE: Semi-supervised federated learning approach for IoT network intrusion detection. Comput. Secur..

[103] Abd Elaziz M., Fares I.A., Dahou A., Shrahili M. (2025). Federated learning framework for IoT intrusion detection using tab transformer and nature-inspired hyperparameter optimization. Front. Big Data.

[104] Ali A., Husain M., Hans P. (2025). Federated learning-enhanced blockchain framework for privacy-preserving intrusion detection in industrial iot. arXiv.

[105] Hossain M.A., Saif S., Islam M.S. (2025). A novel federated learning approach for IoT botnet intrusion detection using SHAP-based knowledge distillation. Complex Intell. Syst..

[106] Ndayipfukamiye T., Ding J., Sarwatt D.S., Philipo A.G., Ning H. (2025). Adversarial Defense in Cybersecurity: A Systematic Review of GANs for Threat Detection and Mitigation. arXiv.

[107] Diab A., Chehade A., Ragusa E., Gastaldo P., Zunino R., Baghdadi A. Intrusion Detection on Resource-Constrained IoT Devices with Hardware-Aware ML and DL. Proceedings of the 2025 IEEE International Conference on Emerging Trends in Engineering and Computing (ETECOM).

[108] Lundqvist J., Kirkeluten T.M., Hadzic A., Pedersen H., Holth J., Johansson M.H., Halkjelsvik M.P.N. (2025). Lightweight Machine Learning Models for Intrusion Detection on IoT Devices. Nor. IKT-Konf. Forsk. Utdanning.

[109] Zhou Y., Chen Y., Rao X., Zhou Y., Li Y., Hu C. (2024). Leveraging Large Language Models and BERT for Log Parsing and Anomaly Detection. Mathematics.

[110] Badhan P.K. (2026). Neuro-symbolic machine learning for lightweight and interpretable IoT edge intrusion detection. Discov. Sens..

[111] Almadhor A., Alsubai S., Hejaili A.A., Klai Z., Bouallegue B., Kovac U. (2025). Designing a neuro-symbolic dual-model architecture for explainable and resilient intrusion detection in IoT networks. Sci. Rep..

[112] Moustafa N., Koroniotis N., Keshk M., Zomaya A.Y., Tari Z. (2023). Explainable Intrusion Detection for Cyber Defences in the Internet of Things: Opportunities and Solutions. IEEE Commun. Surv. Tutor..

[113] Alaskar N.M., Hussain M.S., Almheiri S.J., Khan A., Adnan K.M. (2026). Big Data-Driven Federated Learning Model for Scalable and Privacy-Preserving Cyber Threat Detection in IoT-Enabled Healthcare Systems. Comput. Mater. Contin..

[114] Soomro I.A., Khan H.U.R., Hussain S.J., Iqbal A., Khalid W., Yu H. (2026). SecureDyn-FL: A Robust Privacy-Preserving Federated Learning Framework for Intrusion Detection in IoT Networks. IEEE Trans. Netw. Serv. Manag..

[115] Soomro I.A., Rehman H., Hussain S.J., Latif S., Mujlid H., Mohsin S.M. (2025). ROCHE: A Robust and End-to-End Privacy-Preserving Federated Learning Framework for Intrusion Detection in Industrial Internet of Things. IEEE Internet Things J..

[116] Alqazzaz A. (2026). SecuFL-IoT: An adaptive privacy-preserving federated learning framework for anomaly detection in smart industrial networks. Sci. Rep..

[117] Chen C., Liu J., Tan H., Li X., Wang K.I.K., Li P. (2025). Trustworthy federated learning: Privacy, security, and beyond. Knowl. Inf. Syst..

[118] Rani J.V., Ali H.A.S., Jakka A. IoT Network Intrusion Detection: An Explainable AI Approach in Cybersecurity. Proceedings of the 2023 4th International Conference on Communication, Computing and Industry 6.0 (C216).

[119] Mohale V.Z., Obagbuwa I.C. (2025). A systematic review on the integration of explainable artificial intelligence in intrusion detection systems to enhancing transparency and interpretability in cybersecurity. Front. Artif. Intell..

[120] Gaspar D., Silva P., Silva C. (2024). Explainable AI for Intrusion Detection Systems: LIME and SHAP Applicability on Multi-Layer Perceptron. IEEE Access.

[121] Keshk M., Koroniotis N., Pham N., Moustafa N., Turnbull B., Zomaya A.Y. (2023). An explainable deep learning-enabled intrusion detection framework in IoT networks. Inf. Sci..

[122] Sadhwani S., Navare A., Mohan A., Muthalagu R., Pawar P.M. (2025). IoT-based intrusion detection system using explainable multi-class deep learning approaches. Comput. Electr. Eng..

[123] Nair R. (2023). Unraveling the Decision-making Process Interpretable Deep Learning IDS for Transportation Network Security. J. Cybersecur. Inf. Manag..

[124] Ahmad J., Latif S., Khan I.U., Alshehri M.S., Khan M.S., Alasbali N. (2025). An interpretable deep learning framework for intrusion detection in industrial Internet of Things. Internet Things.

[125] Ahakonye L.A.C., Nwakanma C.I., Lee J.M., Kim D.S. (2024). Machine Learning Explainability for Intrusion Detection in the Industrial Internet of Things. IEEE Internet Things Mag..

[126] Bizzarri A., Yu C.-E., Jalaian B., Riguzzi F., Bastian N.D. (2025). Neurosymbolic AI for network intrusion detection systems: A survey. J. Inf. Secur. Appl..

[127] Sontan A.D., Samuel S.V. (2024). The intersection of Artificial Intelligence and cybersecurity: Challenges and opportunities. World J. Adv. Res. Rev..

[128] Vivo S.D., Obaidat I., Dai D., Liguori P. DDoShield-IoT: A Testbed for Simulating and Lightweight Detection of IoT Botnet DDoS Attacks. Proceedings of the 2024 54th Annual IEEE/IFIP International Conference on Dependable Systems and Networks Workshops (DSN-W).

[129] Kamal H., Mashaly M. (2025). Robust Intrusion Detection System Using an Improved Hybrid Deep Learning Model for Binary and Multi-Class Classification in IoT Networks. Technologies.

[130] Talukder M.A., Islam M.M., Uddin M.A., Hasan K.F., Sharmin S., Alyami S.A. (2024). Machine learning-based network intrusion detection for big and imbalanced data using oversampling, stacking feature embedding and feature extraction. J. Big Data.

[131] Musthafa M.B., Huda S., Kodera Y., Ali M.A., Araki S., Mwaura J. (2024). Optimizing IoT Intrusion Detection Using Balanced Class Distribution, Feature Selection, and Ensemble Machine Learning Techniques. Sensors.

[132] Misrak S.F., Melaku H.M. (2025). Lightweight intrusion detection system for IoT with improved feature engineering and advanced dynamic quantization. Discov. Internet Things.

[133] Heydari S., Mahmoud Q.H. (2025). Tiny Machine Learning and On-Device Inference: A Survey of Applications, Challenges, and Future Directions. Sensors.

[134] Patil R.Y., Bhamare M., Patil Y.H., Bannore A., Chaudhari B.S., Ghorpade S.N., Zennaro M., Paškauskas R. (2024). Chapter 13—Securing TinyML in a connected world. TinyML for Edge Intelligence in IoT and LPWAN Networks.

[135] Huckelberry J., Zhang Y., Sansone A., Mickens J., Beerel P.A., Reddi V.J. (2024). Tinyml security: Exploring vulnerabilities in resource-constrained machine learning systems. arXiv.

[136] Li M., Laiu P., Nichols J.A., Huettel M., Sikkema I., Mathur M. Cognitive IoT and Edge Computing for Intrusion Detection with Federated TinyML. Proceedings of the 2025 IEEE World AI IoT Congress (AIIoT).

[137] Kantharaju V., Suresh H., Niranjanamurthy M., Ansarullah S.I., Amin F., Alabrah A. (2024). Machine learning based intrusion detection framework for detecting security attacks in internet of things. Sci. Rep..

[138] Al-Ajlan M., Ykhlef M. (2024). A Review of Generative Adversarial Networks for Intrusion Detection Systems: Advances, Challenges, and Future Directions. Comput. Mater. Contin..

[139] Hamouda D., Ferrag M.A., Benhamida N., Seridi H., Ghanem M.C. (2024). Revolutionizing intrusion detection in industrial IoT with distributed learning and deep generative techniques. Internet Things.

[140] Qu A., Shen Q., Ahmadi G. (2024). Towards intrusion detection in fog environments using generative adversarial network and long short-term memory network. Comput. Secur..

[141] Gul S., Arshad S., Saeed S.M.U., Akram A., Azam M.A. (2025). WGAN-DL-IDS: An Efficient Framework for Intrusion Detection System Using WGAN, Random Forest, and Deep Learning Approaches. Computers.

[142] Alshehri M.S., Saidani O., Malwi W.A., Asiri F., Latif S., Khattak A.A. (2025). A Hybrid Wasserstein GAN and Autoencoder Model for Robust Intrusion Detection in IoT. Comput. Model. Eng. Sci..

[143] Zhang C., Li J., Wang N., Zhang D. (2025). Research on Intrusion Detection Method Based on Transformer and CNN-BiLSTM in Internet of Things. Sensors.

[144] Yang Y., Peng X. (2025). BERT-based network for intrusion detection system. EURASIP J. Inf. Secur..

[145] Wang Z., Li J., Yang S., Luo X., Li D., Mahmoodi S. (2024). A lightweight IoT intrusion detection model based on improved BERT-of-Theseus. Expert Syst. Appl..

[146] Almadhor A., Alsubai S., Kryvinska N., Hejaili A.A., Ayari M., Bouallegue B. (2025). Evaluating large transformer models for anomaly detection of resource-constrained IoT devices for intrusion detection system. Sci. Rep..

[147] Yin Z., Chen H., Ma H., Hu T., Bai L. (2025). CAEAID: An incremental contrast learning-based intrusion detection framework for IoT networks. Comput. Netw..

[148] Rehman M.U., Bahs H., Kalakoti R. (2026). Incremental Federated Learning for Intrusion Detection in IoT Networks under Evolving Threat Landscape. arXiv.

[149] Mahdi Z.S., Zaki R.M., Alzubaidi L. (2025). A Secure and Adaptive Framework for Enhancing Intrusion Detection in IoT Networks Using Incremental Learning and Blockchain. Secur. Priv..

[150] Kumar R., Swarnkar M. (2025). QuIDS: A Quantum Support Vector machine-based Intrusion Detection System for IoT networks. J. Netw. Comput. Appl..

[151] Kukliansky A., Orescanin M., Bollmann C., Huffmire T. (2024). Network Anomaly Detection Using Quantum Neural Networks on Noisy Quantum Computers. IEEE Trans. Quantum Eng..

[152] Aldhaheri A., Alwahedi F., Ferrag M.A., Battah A. (2024). Deep learning for cyber threat detection in IoT networks: A review. Internet Things Cyber-Phys. Syst..

[153] Ngueajio M.K., Washington G., Rawat D.B., Ngueabou Y. (2023). Intrusion Detection Systems Using Support Vector Machines on the KDDCUP’99 and NSL-KDD Datasets: A Comprehensive Survey. Intelligent Systems and Applications.

[154] Al-Hadhrami Y., Hussain F.K. (2020). Real time dataset generation framework for intrusion detection systems in IoT. Future Gener. Comput. Syst..

[155] Bala R., Nagpal R. (2019). A Review on KDD CUP99 and NSL-KDD Dataset. Int. J. Adv. Res. Comput. Sci..

[156] Divekar A., Parekh M., Savla V., Mishra R., Shirole M. Benchmarking datasets for Anomaly-based Network Intrusion Detection: KDD CUP 99 alternatives. Proceedings of the 2018 IEEE 3rd International Conference on Computing, Communication and Security (ICCCS).

[157] Ashraf W., Masoodi F.S., Khanam A. (2026). Dataset for Evaluating Deep Learning-Based Intrusion Detection. Deep Learning for Intrusion Detection.

[158] Dickson A., Thomas C. (2021). Analysis of UNSW-NB15 Dataset Using Machine Learning Classifiers. Machine Learning and Metaheuristics Algorithms, and Applications.

[159] Luqman M., Zeeshan M., Riaz Q., Hussain M., Tahir H., Mazhar N. (2025). Intelligent parameter-based in-network IDS for IoT using UNSW-NB15 and BoT-IoT datasets. J. Frankl. Inst..

[160] Alsaedi A., Moustafa N., Tari Z., Mahmood A., Anwar A. (2020). TON_IoT Telemetry Dataset: A New Generation Dataset of IoT and IIoT for Data-Driven Intrusion Detection Systems. IEEE Access.

[161] Ismail S., Dandan S., Qushou A. (2025). Intrusion Detection in IoT and IIoT: Comparing Lightweight Machine Learning Techniques Using TON_IoT, WUSTL-IIOT-2021, and EdgeIIoTset Datasets. IEEE Access.

[162] Hajjouz A., Avksentieva E. (2024). Optimizing Intrusion Detection for DoS, DDoS, and Mirai Attacks Subtypes Using Hierarchical Feature Selection and CatBoost on the CICIoT2023 Dataset. Data Metadata.

[163] Meena G., Indian A. (2025). IDS-IoT: Intrusion Detection System for the Internet of Things Using Enhanced Long-Short Term Memory. Artif. Intell. Appl..

[164] Thereza N., Ramli K. Development of Intrusion Detection Models for IoT Networks Utilizing CICIoT2023 Dataset. Proceedings of the 2023 3rd International Conference on Smart Cities, Automation & Intelligent Computing Systems (ICON-SONICS).

[165] Alosaimi S., Almutairi S.M. (2023). An Intrusion Detection System Using BoT-IoT. Appl. Sci..

[166] Chua T.-H., Salam I. (2023). Evaluation of Machine Learning Algorithms in Network-Based Intrusion Detection Using Progressive Dataset. Symmetry.

[167] Hossain M.D., Mahin M.J.H., Khan M.R., Akther M., Habib M.A. Hybrid Deep Learning and Ensemble Methods for Dependable IoT Intrusion Detection. Proceedings of the IEEE 7th International Conference on Sustainable Technologies For Industry 5.0 (STI).

[168] Schmidt H., Sutterfield G., Farnell C. Addressing Cybersecurity Data and Workforce Scarcity with TROY: Testbed for Resilient Operational sYstems. Proceedings of the IEEE Design Methodologies Conference (DMC).

[169] Sharma H., Kumar P., Sharma K. (2025). Intelligent Time Series Analysis for Intrusion Detection in the Internet of Things: A Generative-Adversarial-Network-Enhanced Convolutional-Neural-Network–Long-Short-Term-Memory Framework Using Signal Features. Intell. Comput..

[170] Putro I.H., Ahmad T., Ijtihadie R.M. (2025). Enhancing MQTT Intrusion Detection in IoT Using Machine Learning and Feature Engineering. IEEE Open J. Commun. Soc..

[171] Nair A.R., Praveen I. (2026). A Stacking-Enhanced Voting Ensemble Model for Network Intrusion Detection and for Security of Internet of Things. Data Science and Applications.

